# Cytoplasmic NEAT1 Suppresses AML Stem Cell Self‐Renewal and Leukemogenesis through Inactivation of Wnt Signaling

**DOI:** 10.1002/advs.202100914

**Published:** 2021-10-05

**Authors:** Huiwen Yan, Zhi Wang, Yao Sun, Liangding Hu, Pengcheng Bu

**Affiliations:** ^1^ Key Laboratory of RNA Biology Key Laboratory of Protein and Peptide Pharmaceutical Institute of Biophysics Chinese Academy of Sciences Beijing 100101 China; ^2^ Department of Hematopoietic Stem Cell Transplantation the Fifth Medical Center of Chinese PLA General Hospital Beijing 100071 China; ^3^ Center for Excellence in Biomacromolecules Chinese Academy of Sciences Beijing 100101 China; ^4^ College of Life Sciences University of Chinese Academy of Sciences Beijing 100049 China

**Keywords:** acute myeloid leukemia, nuclear paraspeckle assembly transcript 1, translocation, ubiquitination, Wnt

## Abstract

As an essential component of paraspeckles, nuclear paraspeckle assembly transcript 1 (NEAT1) localizes in the nucleus, promoting progression of various malignant solid tumors. Herein, an adverse effect of NEAT1 is reported, showing that the short isoform, NEAT1_1 suppresses acute myeloid leukemia (AML) development. NEAT1_1 is downregulated in leukemia stem cells (LSCs) and its decreased expression correlates with recurrence in AML patients. It is demonstrated that NEAT1_1 suppresses leukemogenesis and LSC function but is dispensable for normal hematopoiesis. Mechanistically, NEAT1_1 is released from the nucleus into the cytoplasm of AML cells, regulated by transcription factor C/EBP*β* and nuclear protein NAP1L1. Cytoplasmic NEAT1_1 interacts with Wnt component DVL2 and E3 ubiquitin ligase Trim56, facilitates Trim56‐mediated DVL2 degradation, and thus suppresses Wnt signaling. Collectively, the findings show NEAT1_1 is translocated from the nucleus to the cytoplasm and acts as a tumor suppressor in AML.

## Graphical abstract

Nuclear paraspeckle assembly transcript 1 (NEAT1) is an important component of paraspeckles, shown to localize to the nucleus and act as an oncogene, promoting the progression of many types of solid tumors. However, this work reveals that NEAT1 functions as a tumor suppressor in acute myeloid leukemia (AML) by translocating to cytoplasm in AML cells, and manipulating Wnt signaling.

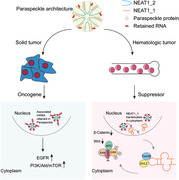

## 1 Introduction

Acute myeloid leukemia (AML) is a fatal form of hematopoietic malignancy,with 5‐year survival rate of adult patients only 24%.^[^ ^1^ ^]^ Despite the progress of therapeutic approaches such as allogeneic hematopoietic stem cell transplantation (allo‐HSCT) and chimeric antigen receptor (CAR) T cell treatment, the survival of AML patients has not been significantly improved. Up to 50% AML patients end up relapsing after receiving allo‐HSCT, while CAR‐T cell treatment frequently fails due to lack of a specific AML antigen,^[^ ^2^ ^]^ antigen loss, or failure of CAR‐T cell maintenance in AML patients.^[^ ^3^ ^]^ Thus, it is important to better understand the molecular basis of AML progression in order to develop more effective AML treatment strategies.

AML is characterized by uncontrolled expansion and block in differentiation of leukemia stem cells (LSCs),^[^ ^4^ ^]^ which are responsible for AML initiation, progression, and relapse.^[^ ^5^ ^]^ LSC accumulation is caused by rearrangements, mutations, or aberrant expression of signaling nodes^[^ ^6^ ^]^ that regulate LSC self‐renewal and differentiation.^[^ ^7^ ^]^ Wnt/*β*‐catenin signaling is one of such signaling nodes, notably critical for LSC maintenance, with dishevelled 2 (DVL2) functioning as a principal Wnt component. In the absence of Wnt ligands, *β*‐catenin is degraded by the APC/Axin/CK1*α*/GSK3*β* degradation complex‐mediated ubiquitin‐proteasome pathway. Upon Wnt ligands binding to Fzd receptors, DVL2 disassembles the degradation complex, thus stabilizing *β*‐catenin.^[^ ^8^ ^]^ While accumulating evidence shows the importance of *β*‐catenin in regulating LSC function, little is known about the role and regulatory mechanism of DVL2 in LSC of AML.^[^ ^9^ ^]^

Long non‐coding RNAs (lncRNAs) play crucial roles in various biological processes such as tumorigenesis. Nuclear paraspeckle assembly transcript 1 (NEAT1) is one of the most well‐studied lncRNAs, which contains a short isoform, NEAT1_1 (3.7kb),^[^ ^10^ ^]^ and a long isoform, NEAT1_2 (23kb).^[^ ^11^ ^]^ NEAT1 is located in the nucleus and acts as an essential component of paraspeckles.^[^ ^11^ ^]^ Since first being identified in 2007, NEAT1 has been shown to be upregulated in the majority of solid tumors,^[^ ^12^ ^]^ such as breast cancer,^[^ ^13^ ^]^ prostate cancer,^[^ ^14^ ^]^ colorectal cancer,^[^ ^15^ ^]^ lung cancer,^[^ ^16^ ^]^ ovarian cancer, and pancreatic cancer, and promotes tumor development, or works as a prognostic maker.^[^ ^17^ ^]^ Here, surprisingly, we show that NEAT1 is downregulated in AML and its underexpression is associated with enhanced relapse. Our functional study revealed that NEAT1_1 acts as a tumor suppressor, inhibiting leukemogenesis and LSC self‐renewal by inactivating Wnt signaling in AML cell cytoplasm.

## 2 Results

### 2.1 NEAT1 Is Downregulated in LSCs

**Figure 1:**
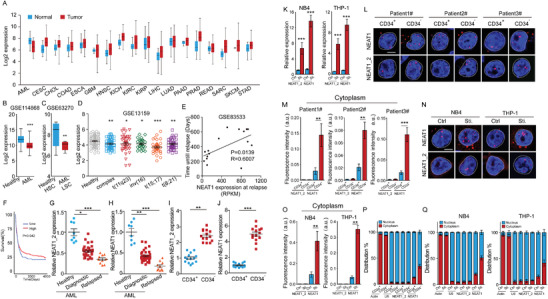
NEAT1 is downregulated in acute myeloid leukemia stem cells. A) NEAT1 expression of AML along with 17 types of the most common solid tumors and their paired normal tissues in the TCGA database. B) NEAT1 expression in bone marrow from 194 AML patients and 20 healthy donors (GSE114868). C) NEAT1 expression in LSCs from 15 AML patients and HSCs from 7 healthy donors (GSE63270). D) NEAT1 expression in bone marrow or peripheral blood from AML patients bearing various cytogenetic aberrations with those from healthy donors (GSE13159). E) Pearson's correlation between NEAT1 expression and days to relapse of AML patients (GSE83553). F) Kaplan–Meier plots of overall survival of AML patients in the TCGA database. G,H) RT‐qPCR showing NEAT1_2 (G) and total NEAT1 (H) expression in bone marrow cells from healthy donors (*n* = 8), diagnostic (*n* = 30), and relapsed (*n* = 10) AML patients. I,J) RT‐qPCR showing NEAT1_2 (I) and total NEAT1 (J) expression in CD34^+^ LSCs and CD34^−^ AML cells isolated from AML (*n* = 14) patients. K) RT‐qPCR showing NEAT1_2 and total NEAT1 expression in NB4 (Ctrl) and ATRA‐induced differentiated (Sti) cells, as well as THP‐1 (Ctrl) and PMA‐induced differentiated (Sti) cells. L,M) Cellular location of NEAT1 and NEAT1_2 in CD34+ LSCs and CD34− AML cells isolated from AML patients. Representative RNA FISH images (L) and cytoplasmic RNA FISH signals in each cell (M) were shown. 20 CD34+ LSCs and 22 CD34− AML cells were analyzed. N,O) Cellular location of NEAT1 and NEAT1_2 in NB4 (Ctrl) and ATRA‐induced differentiated (Sti) cells, as well as THP‐1 (Ctrl) and PMA‐induced differentiated (Sti) cells. Representative RNA FISH images (N) and cytoplasmic RNA FISH signals in each cell (O) were shown. 32 parent and 35 differentiated cells were analyzed. P) RT‐qPCR showing total NEAT1 and NEAT1_2 levels in cytoplasmic and nuclear fractions in CD34^+^ LSCs and CD34^−^ AML cells (*n* = 14). Actin and U6 are cytoplasmic and nuclear markers, respectively. Q) RT‐qPCR showing total NEAT1 and NEAT1_2 levels in cytoplasmic and nuclear fractions in NB4 (Ctrl) and ATRA‐induced differentiated (Sti) cells, as well as THP‐1 (Ctrl) and PMA‐induced differentiated (Sti) cells. Data represent the mean ± standard deviation (SD) in (K) and mean ± standard error of the mean (S.E.M.) in other figures. *P*‐value was calculated based on log‐rank test in (F) and a Student's *t*‐test in other figures. *p < 0.05, **p < 0.01, and ***p < 0.001. Scale bar: 5 µm. AML: acute myeloid leukemia, BLCA: bladder urothelial carcinoma, CESC: cervical squamous cell carcinoma, CHOL: cholangiocarcinoma, COAD: colon adenocarcinoma, ESCA: esophageal carcinoma, GBM: glioblastoma multiforme, HNSC: head and neck squamous cell carcinoma, KICH: kidney chromophobe, KIRC: kidney renal clear cell carcinoma, KIRP: Kidney renal papillary cell carcinoma, LIHC: liver hepatocellular carcinoma, LUAD: lung adenocarcinoma, PAAD: Pancreatic adenocarcinoma, PRAD: Prostate adenocarcinoma, READ: rectal adenocarcinoma, SARC: sarcoma, SKCM: skin cutaneous melanoma, STAD: stomach adenocarcinoma.

To evaluate the potential function of NEAT1 in AML, we first analyzed NEAT1 expression in AML along with 17 types of the most common solid tumors in the cancer genome atlas (TCGA) datasets. Consistent with previous reports, NEAT1 expression is elevated in solid tumors in contrast to the paired normal controls. Conversely, we observed that NEAT1 was significantly downregulated in the AML samples compared to the healthy controls (**Figure** **1**A). NCBI Gene Expression Omnibus (GEO) datasets further showed that NEAT1 was downregulated in AML cells, particularly in LSCs (Figure 1B,C). In addition, NEAT1 downregulation occurs in AML cells from various subtypes of AML patients (Figure 1D). Analysis of the prognostic value of NEAT1 expression in AML revealed that high NEAT1 expression was positively correlated with duration until relapse and overall survival of the AML patients (Figure 1E,F). Thus, NEAT1 is downregulated in AML, contrary to previous observations that NEAT1 is an oncogene and upregulated in various solid tumors.

NEAT1 has two isoforms, a shorter NEAT1_1 isoform which completely overlaps with the 5′ end of the longer NEAT1_2 isoform.^[^ ^18^ ^]^ To investigate which NEAT1 isoform is downregulated in AML, we collected bone marrow samples from diagnostic and relapsed AML patients and healthy donors. NEAT1 levels were measured by RT‐qPCR using primers for total NEAT1 (NEAT1_1 and NEAT1_2) and specific to NEAT1_2 (Figure S1A, Supporting Information). We observed that both NEAT1 and NEAT1_2 expression were decreased in AML diagnostic samples and further decreased in AML relapsed samples, compared to the healthy controls (Figure 1G,H). LSCs reside in CD34^+^ AML cells, responsible for occurrence, therapeutic resistance, and relapse of AML. We then isolated CD34^+^ LSCs from AML patients. RT‐qPCR showed that both NEAT1 and NEAT1_2 were downregulated in LSCs compared to the CD34^−^ AML cells (Figure 1I,J). Consistently, both NEAT1 and NEAT1_2 were also downregulated in human AML cell THP‐1 and NB4 compared to the differentiated cells induced by phorbol myristate acetate (PMA) or All‐Trans retinoic Acid (ATRA), respectively (Figure 1K). We further performed RNA fluorescence in situ hydroazidation (RNA‐FISH) on the CD34^+^ LSCs and CD34^−^ AML cells using probes targeting the 5′ and 3′ region of NEAT1 to measure total NEAT1 or NEAT1_2, respectively (Figure S1A, Supporting Information). Consistently, NEAT1 and NEAT1_2 expression was elevated in CD34^−^ AML cells compared to CD34^+^ LSCs (Figure 1L,M; Figure S1B, Supporting Information). Similar results were observed in THP‐1 and NB4 and the differentiated cells induced by PMA and ATRA, respectively (Figure 1N,O; Figure S1C, Supporting Information). Taken together, NEAT1 was indeed downregulated in AML cells, and particularly in LSCs.

### 2.2 NEAT1_1 Is Elevated in AML Cell Cytoplasm

Previous studies showed that NEAT1 was localized in nucleus to maintain the structural integrity of the paraspeckles.^[^ ^19^ ^]^ Interestingly, our RNA‐FISH revealed that unlike NEAT1_2, which was restricted in the nucleus, a small proportion of NEAT1 (including NEAT1_1 and NEAT1_2) existed in the cytoplasm of CD34^+^ LSCs (Figure 1L,M). Moreover, NEAT1 levels were significantly elevated in the cytoplasm of CD34^−^ AML cells, in contrast to NEAT1_2 which remained in the nucleus of CD34^−^ AML cells (Figure 1L,M). Similarly, we observed that the total NEAT1 was significantly increased in the cytoplasm of differentiated cells compared to the parent THP‐1 and NB4 cells, while NEAT1_2 was only localized to the nucleus in either THP‐1 and NB4 cells (Figure 1N,O). These results indicate that the short isoform NEAT1_1 localizes to both the nucleus and cytoplasm of LSCs. Moreover, cytoplasmic NEAT1_1 is significantly elevated in non‐LSCs compared to LSCs. To further probe these results, we isolated the cytoplasmic and nuclear fraction of the cells, using actin and U6 as controls, respectively. RT‐qPCR further confirmed that total NEAT1, but not NEAT1_2 was enriched in the cytoplasm of CD34^−^ AML cells and the differentiated cells derived from THP‐1 and NB4 cells (Figure 1P,Q). We further performed Northern blot for NEAT1 and NEAT1 levels in cytoplasmic and nuclear fractions isolated from NB4 cells and bone marrow cells isolated from two AML patients. Consistent with RT‐qPCR and RNA‐FISH results, we found that NEAT1_1 existed in both cytoplasm and nucleus of the AML cells. In contrast, NEAT1_2 only remained in the nuclear fractions (Figure S1D, Supporting Information). Thus, NEAT1_1 is elevated in CD34^−^ AML cell cytoplasm, suggesting that NEAT1_1 may have a distinct function beyond paraspeckle assembly.

### 2.3 NEAT1_1 Suppresses Human AML Cell Growth and LSC Function

**Figure 2:**
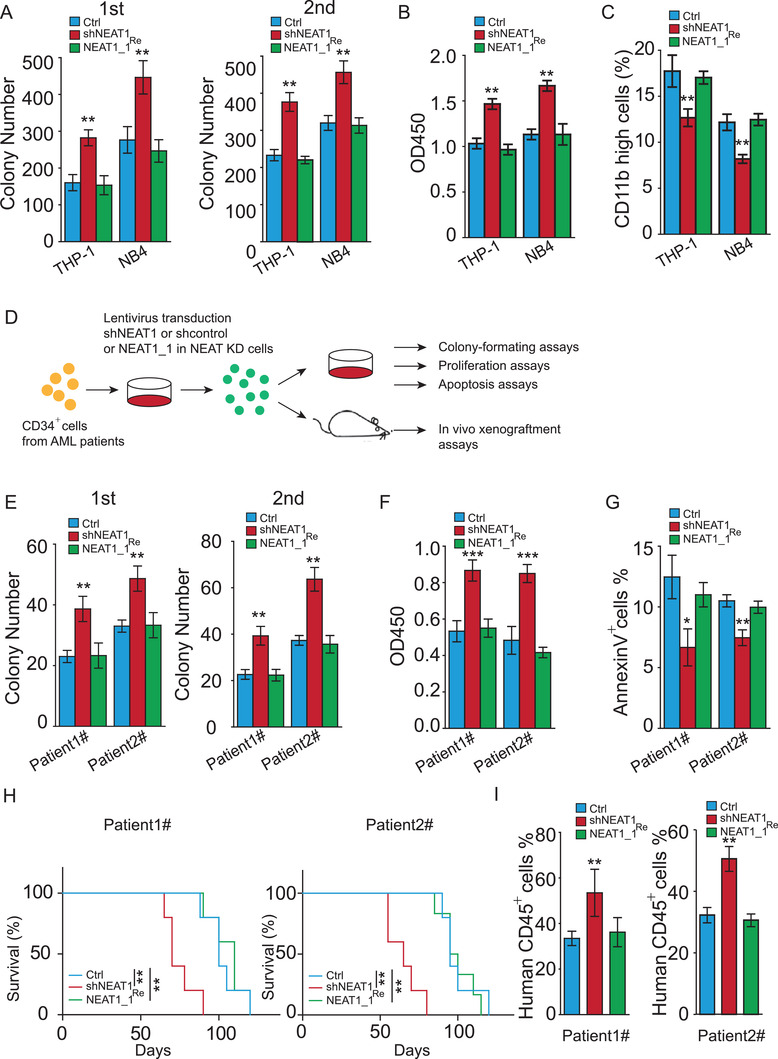
NEAT1_1 suppresses human AML maintenance and human LSC function. A–C) Colony formation (A), proliferation (B), and differentiation (C) of NB4 and THP1 cells when total NEAT1 was knocked down (shNEAT1) or NEAT1_1 was restored (NEAT1_1^Re^). D) Experimental scheme for (E–I). E–G) Colony formation (E), proliferation (F), and apoptosis (G) of primary lin^−^CD34^+^ cells from diagnostic (1#) and relapsed (2#) AML patients when total NEAT1 was knocked down (shNEAT1) or NEAT1_1 was restored (NEAT1_1^Re^). H) Kaplan–Meier survival plots of receipt mice transplanted with primary lin^−^CD34^+^ cells from diagnostic (1#) and relapsed (2#) AML patients when total NEAT1 was knocked down (shNEAT1) and NEAT1_1 was restored (NEAT1_1^Re^) (*n* = 5, each group). I) Percentage of human CD45^+^ cells in receipt mice transplanted with primary CD34^+^ cells from diagnostic (1#) and relapsed (2#) AML patients when total NEAT1 was knocked down (shNEAT1) and NEAT1_1 was restored (NEAT1_1^Re^) (*n* = 3). The data represent the mean ± S.E.M. in (I) and mean ± SD in the other figures. The *p*‐values were calculated based on log‐rank test in (H) and a Student's *t*‐test in other figures. *p < 0.05; **p < 0.01; ***p < 0.001.

To understand NEAT1_1 function in human AMLs, we knocked down total NEAT1 in NB4 and THP‐1 cells using two independent shRNAs (Figure S2A, Supporting Information). The total NEAT1‐depleted cells were then forcedly expressed with NEAT1_1, and the efficiency of NEAT1 knockdown and rescue was validated by RT‐qPCR (Figure S2B, Supporting Information). We observed that knockdown of total NEAT1 resulted in increased colony formation, proliferation, and decreased differentiation, which was fully rescued by forced expression of NEAT1_1 (**Figure** **2**A–C). We then freshly isolated lin^–^CD34^+^ LSCs from diagnostic and relapsed AML patients. Similarly, we knocked down total NEAT1 and rescued with NEAT1_1 in the primary LSCs (Figure S2C, Supporting Information). We then examined the capability of colony formation, proliferation, and apoptosis in vitro and leukemia reconstitution in vivo (Figure 2D). Similar to the observation in the NB4 and THP‐1 cells, total NEAT1 knockdown in LSCs significantly increased colony formation, enhanced proliferation and suppressed apoptosis, which was abrogated by restoring NEAT1_1 expression (Figure 2E–G). Immunodeficient NSG mice implanted with LSCs from diagnostic or relapsed AML patients demonstrated that total NEAT1 knockdown significantly accelerated AML progression and shortened the AML‐bearing mice survival time, while rescue with NEAT1_1 efficiently rescued the phenotypes resulted from NEAT1 knockdown (Figure 2H). In addition, we observed that total NEAT1 knockdown significantly enhanced the engraftment of donor cells (human CD45^+^ cells) in NSG mice, which was rescued by restoring NEAT1_1 in total NEAT1 knockdown cells (Figure 2I). Together, the data indicate that NEAT1_1 suppresses leukemogenesis of human AML by regulation of LSCs.

### 2.4 NEAT1_1 Suppresses Murine Leukemogenesis and LSC Function

**Figure 3:**
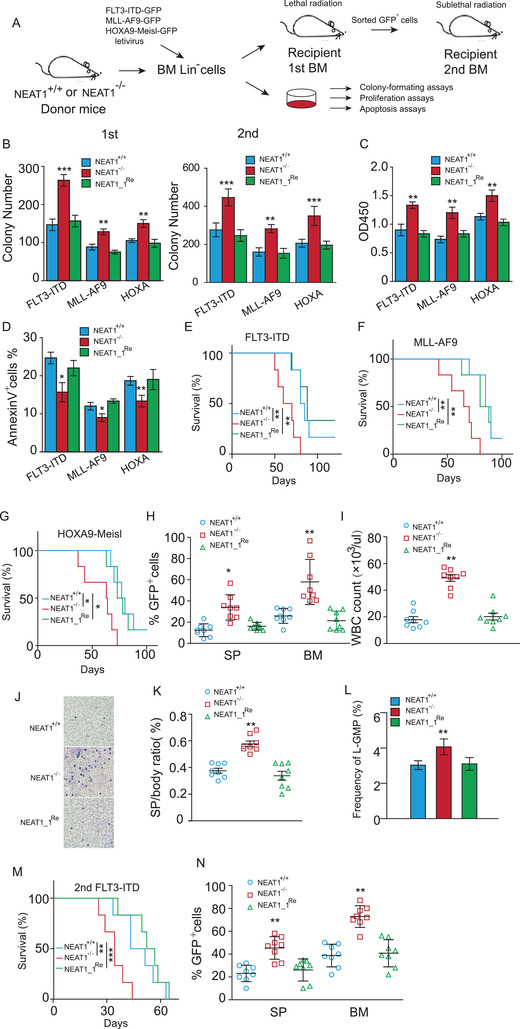
NEAT1_1 suppresses murine AML development and LSC function. A) Experimental scheme for FLT3‐ITD‐, MLL‐AF9‐, or HOXA‐Meis1‐induced murine AML model and functional assays. Lin^−^ cells were isolated from wild‐type (NEAT1^+/+^) and NEAT1 knockout (NEAT1^−/−^) murine BM, and restored with NEAT1_1 (NEAT1_1^Re^). B–D) Colony (B), proliferation (C), and apoptosis (D) of primary lin^−^ cells from murine bone marrow after transducing with FLT3‐ITD‐GFP, MLL‐AF9‐GPF, or HOXA‐Meis1‐GFP lentivirus. E–G) Kaplan–Meier survival plots of recipient mice transplanted with FLT3‐ITD‐GFP‐ (E), MLL‐AF9‐GPF‐ (F), or HOXA‐Meis1‐GFP‐transduced (G) primary lin^−^ cells (*n* = 6, each group). H) Percentage of GFP positive cells in spleen and bone marrow of recipient mice (*n* = 8, each group). I) White blood cell (WBC) count in peripheral blood (PB) of recipient mice (*n* = 8, each group). J) Representative images of Wright–Giemsa staining of peripheral blood (PB) from recipient mice. K) Spleen weight of recipients (*n* = 8, each group). L) Percentage of L‐GMP cells in recipient mice (*n* = 8, each group). M) Kaplan–Meier survival plots of recipient mice that received secondary bone marrow transplantation (*n* = 6, each group). N) Percentage of GFP positive cells in the spleen and bone marrow from recipient mice that received secondary bone marrow transplantation (*n* = 8, each group). The data represent the mean ± SD in (B–D) and mean ± S.E.M. in (H,I,K,L,N). The *p*‐values were calculated based on log‐rank test in (E–G and M) and a Student's *t*‐test in other figures. *p < 0.05; **p < 0.01; ***p < 0.001.

To investigate whether NEAT1_1 influences murine leukemogenesis, we used NEAT1 knockout (NEAT1^−/−^) mice, in which both NEAT1_1 and NEAT1_2 were deleted.^[^ ^20^ ^]^ Lin^−^ bone marrow cells were isolated from mice and restored with NEAT1_1 (NEAT1_1^Re^). We subsequently infected the cells with MLL‐AF9‐GFP,^[^ ^21^ ^]^ HOXA9‐Meis1‐GFP^[^ ^22^ ^]^ or FLT3‐ITD‐GFP,^[^ ^23^ ^]^ the most prevalent rearranged fusion or mutant genes widely used for AML model construction (**Figure** **3**A). We observed that total NEAT1 knockout significantly promoted MLL‐AF9‐, HOXA9‐Meis1‐ and FLT3‐ITD‐mediated cell immortalization, showing increased colony formation and proliferation, and decreased apoptosis (Figure 3B–D). In contrast, restorage of NEAT1_1 fully rescued total NEAT1 deletion‐enhanced cell immortalization (Figure 3B–D). To evaluate the role of NEAT1_1 in leukemogenesis in vivo, we transplanted the MLL‐AF9‐, HOXA9‐Meis1‐, and FLT3‐ITD‐transformed bone marrow cells into lethally irradiated recipient mice (Figure 3A). We observed that total NEAT1 deletion significantly accelerated AML progression and shortened mice survival in all three AML models (Figure 3E–G), while restoring NEAT1_1 efficiently rescued the phenotype (Figure 3E–G). We then performed another bone marrow transplantation assay using the FLT3‐ITD‐induced AML model and euthanized the mice simultaneously when one mouse became moribund. We observed that compared to the mice transplanted with wild‐type bone marrow, those with NEAT1^−/−^ bone marrow cells exhibited an increased donor cell (GFP positive cell) ratio in the spleen and bone marrow, resulting in an increased white blood cell count, spleen size, and immature blast cell population in peripheral blood (Figure 3H–K). The phenomena were rescued by restoring NEAT1_1 in NEAT1^−/−^ bone marrow cells (Figure 3H–K). Thus, loss of NEAT1_1 promotes murine leukemogenesis.

We next evaluated the effect of NEAT1_1 on LSC activity. The bone marrow transplantation assay showed that total NEAT1 knockout increased the frequency of leukemic granulocyte‐monocyte progenitor (L‐GMP, lin^−^Sca‐1^−^c‐kit^+^CD34^+^CD127^−^CD16/32^hi^) in the receipt bone marrow compared to the wild‐type controls. In contrast, the frequency of L‐GMP did not change much when NEAT1_1 was forcedly expressed in NEAT1^−/−^ bone marrow cells (Figure 3L). Moreover, mice that received a secondary transplantation of NEAT1^−/−^ bone marrow cells developed AML and died even earlier compared to mice transplanted with wild‐type or NEAT1_1 rescued‐bone marrow cells (Figure 3M). In addition, increased donor cells (GFP positive cells) were also observed in the spleen and bone marrow of the mice that received a secondary transplantation of NEAT1^−/−^ bone marrow cells, which was rescued by restoring NEAT1_1 (Figure 3N). Together, the data suggest that NEAT1_1 is critical for LSC repopulation.

### 2.5 NEAT1 Is Dispensable for Normal Hematopoiesis

**Figure 4:**
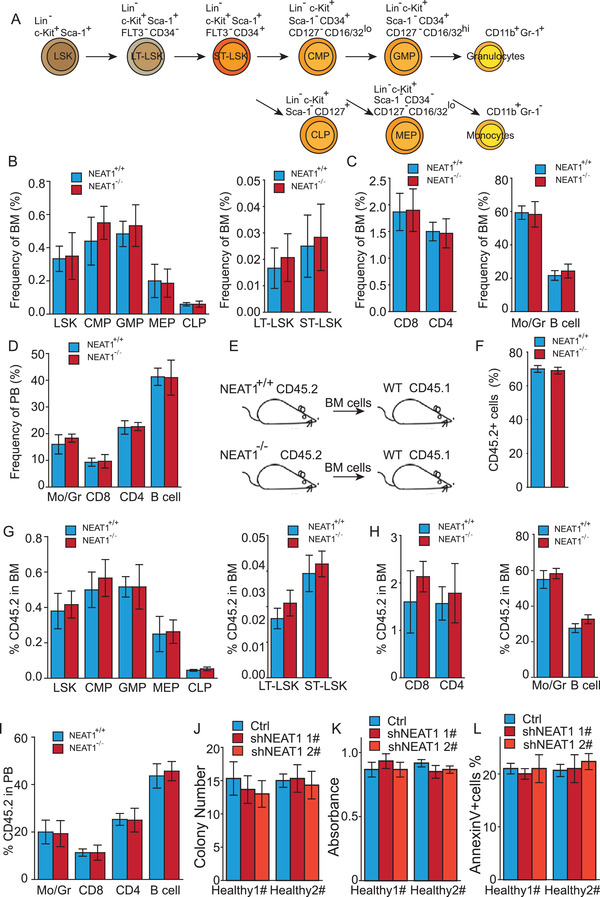
NEAT1 is dispensable for normal hematopoiesis. A) Origins and cell surface markers used to identify mouse hematopoietic stem cell and myeloid progenitor lineages. B–D) Percentage of the population of stem cells, progenitors, and mature lineage cells in the bone marrow and spleen of 8‐week‐old wildtype (NEAT1^+/+^) and NEAT1 knockout (NEAT1^−/−^) mice (*n* = 6, each group). E) Experimental scheme of the bone marrow transplantation assays for (F–I). F) Percentage of CD45.2 cells in the bone marrow. G–I) Percentage of different populations of stem cells, progenitors, and mature lineage cell of CD45.2^+^ cells in recipient mice (*n* = 5, each group). J–L) Colony formation (J), proliferation (K), and apoptosis (L) of human lin^−^CD34+ cells and the cells with NEAT1‐knocked down. Data represent the mean ± SD in (J–L) and mean ± S.E.M. in other figures.

Next, we evaluated whether NEAT1 is dispensable for normal hemopoiesis. We first isolated Lin^–^ cells from wild‐type (NEAT1^+/+^) and NEAT1 knockout (NEAT1^−/−^) mice and examined the capability of colony formation, proliferation, and apoptosis, which showed comparable cellular behavior between wild‐type and NEAT1‐deficient Lin^−^ cells (Figure S3A‐C, Supporting Information). To evaluate the effect of NEAT1 on murine hemopoiesis in vivo, we analyzed the frequency of Lin^–^Sca‐1^+^c‐Kit^+^ (LSK) cells, progenitors, and mature lineage cells in NEAT1^+/+^ and NEAT1^−/−^ mice at 8 weeks old and 1 year old (**Figure** **4**A). We observed that NEAT1^−/−^ and NEAT1^+/+^ mice showed comparable ratio of LSK cells, granulocyte monocyte progenitors (GMP), common myeloid progenitors (CMP), megakaryocyte erythrocyte progenitors (MEP), common lymphoid progenitors (CLP), T cells, and B cells when the mice were at 8 weeks old (Figure 4B–D). Moreover, analysis of the aged mice (1 year old) showed that NEAT1 knockout also did not cause significant changes in the composition of f LSK cells and the progenies (Figure S3D–F, Supporting Information). Furthermore, we performed bone marrow transplantation with NEAT1^+/+^ and NEAT1^−/−^ cells. The recipient mice exhibited equivalent chimerism in all hematopoietic cells at the early (8 weeks) and late (8 months) time point after transplantation (Figure 4E–I; Figure S3G–I, Supporting Information). Consistently, competitive serial transplantation assays showed that NEAT1 knockout did not affect self‐renewal capacity of HSCs and progenitor cells in vivo (Figure S3J–L, Supporting Information). Together, the data suggest that NEAT1 does not influence normal murine hematopoietic stem cell (HSC) functions.

We then examined the effect of NEAT1_1 on human HSC functions. We knocked down total NEAT1 in human bone marrow‐derived lin^−^CD34^+^ cells, and validated by RT‐qPCR (Figure S3M, Supporting Information). Here, NEAT1 knockdown cells exhibited comparable capability of colony formation, proliferation, and apoptosis with the control cells (Figure 4J–L). Taken together, our results indicate that NEAT1 depletion does not influence HSC function.

### 2.6 C/EBP*β* and NAP1L1 Co‐operate to Elevate NEAT1_1 in AML Cells Cytoplasm

**Figure 5:**
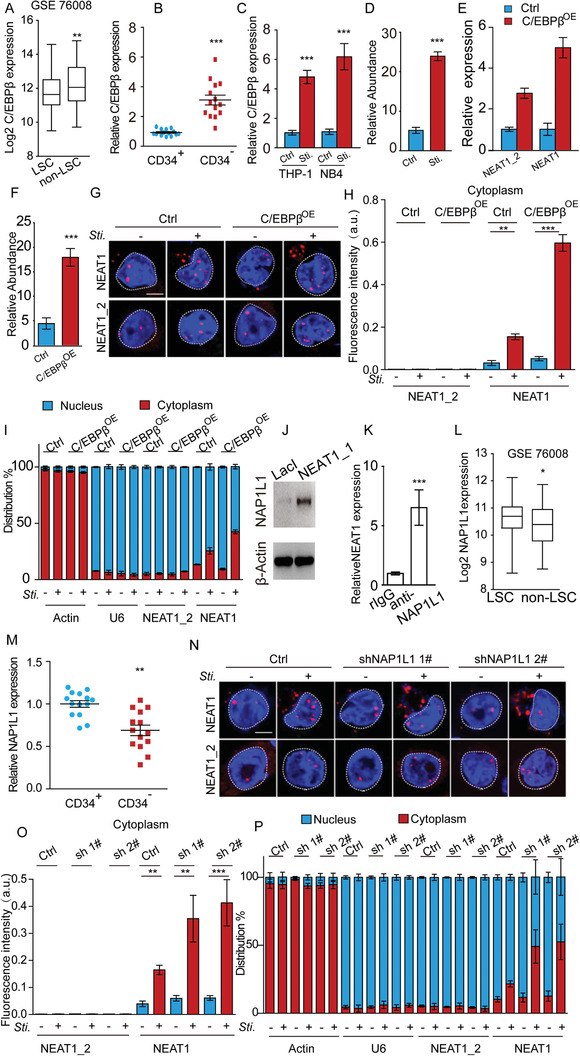
Co‐operation of C/EBP*β* and NAP1L1 upregulates NEAT1_1 in AML cell cytoplasm. A) C/EBP*β* expression in LSCs and non‐LSCs in GEO dataset (GSE76008). B) RT‐qPCR showing C/EBP*β* expression in CD34^+^ LSCs and CD34^−^ AML cells isolated from AML patients (*n* = 14). C) RT‐qPCR showing C/EBP*β* expression in THP‐1, NB4, and PAM‐ or ATRA‐induced differentiated cells. D) ChIP‐qPCR showing enrichment of C/EBP*β* binding to the NEAT1 promoter in NB4 and ATRA‐induced differentiated cells. Signals were normalized with Actin (input). The fold changes were calculated by normalizing with immunoglobulin G (IgG) control antibody. E) RT‐qPCR showing total NEAT1 and NEAT1_2 expression levels in the control and C/EBP*β* ^OE^ cells. F) ChIP‐qPCR showing enrichment of C/EBP*β* binding to the NEAT1 promoter in the C/EBP*β* ^OE^ cells relative to control cells. G,H) Cellular location of NEAT1 and NEAT1_2 in NB4 cells (Ctrl) and NB4 cells with C/EBP*β*‐enforced expression (C/EBP*β* ^OE^). Representative RNA FISH images (G) and cytoplasmic RNA FISH signals in each cell (H) are shown. 35 control cells and 32 C/EBP*β* ^OE^ cells were analyzed. I) RT‐qPCR showing total NEAT1 and NEAT1_2 levels in cytoplasmic and nuclear fractions in NB4 cells (Ctrl) and NB4 cells with C/EBP*β*‐enforced expression (C/EBP*β* ^OE^). Actin and U6 are cytoplasmic and nuclear markers, respectively. J) Western blot following RNA pulldown showing NEAT1_1 interacting with NAP1L1. K) RNA immunoprecipitation (RIP) assay showing NEAT1_1 interacting with NAP1L1. L) NAP1L1 expression in LSCs and non‐LSCs in GEO dataset (GSE 76008). M) RT‐qPCR showing NAP1L1 expression in CD34^+^ LSCs and CD34^−^ AML cells isolated from AML patients (*n* = 14). N,O) Cellular location of NEAT1 and NEAT1_2 in NB4 cells (Ctrl) and NB4 cells with NAP1L1‐knocked down (sh 1# and sh 2#). Representative RNA FISH images (N) and cytoplasmic RNA FISH signals in each cell (O) are shown. 29 control cells and 30 of each shRNA‐mediated NAP1L1 knockdown cells were analyzed. P) RT‐qPCR showing total NEAT1 and NEAT1_2 levels in cytoplasmic and nuclear fractions in NB4 cells (Ctrl) and NB4 cells with NAP1L1‐knocked down (sh 1# and sh 2#). Actin and U6 are cytoplasmic and nuclear markers, respectively. Data represent the mean ± S.E.M. in (A,B,H,I,L,O,P) and mean ± SD in (C,D,E,F,K). *P*‐value was calculated based on a Student's *t*‐test. *p < 0.05; **p < 0.01; ***p < 0.001. Scale bar: 5 µm.

Initially, we showed that the NEAT1_1 level increased in CD34^−^ AML cells compared to CD34^+^ LSCs, so as in differentiated cells compared to the parent NB4 and THP‐1 cells (Figure 1L–Q). To understand the potential mechanism, we analyzed the 2 kb sequence of the NEAT1 promoter in the PROMO database^[^ ^24^ ^]^ and found that 5 transcription factors including Fos, Jun, Usf1, YY1, and CCAAT enhancer binding protein beta (C/EBP*β*) can potentially bond to the NEAT1 promoter (Figure S4A, Supporting Information). We evaluated the expression of the transcription factors in NB4 cells and the differentiated cells induced by ATRA, and found that only C/EBP*β* was upregulated in the differentiated cells compared to the parent NB4 cells (Figure S4B, Supporting Information). Analysis of GEO database (GSE76008) revealed that C/EBP*β* was upregulated in non‐LSC cells compared with LSC cells (**Figure** **5**A). Therefore, we further measured C/EBP*β* expression in CD34^+^ LSCs and CD34^−^ AML cells, finding that C/EBP*β* was upregulated in CD34^−^ AML cells (Figure 5B). Consistently, we observed that C/EBP*β* was upregulated in the differentiated cells compared with the parent THP‐1 and NB4 cells (Figure 5C; Figure S4B, Supporting Information). C/EBP*β* is an important tumor suppressor and acts by regulating myeloid differentiation.^[^ ^25^ ^]^ ChIP‐qPCR confirmed that C/EBP*β* bound to the NEAT1 promoter, and enforced expression of C/EBP*β* enriched C/EBP*β* binding to NEAT1 promoter and upregulated NEAT1 expression in AML cells (Figure 5D–F). Furthermore, enforced expression of C/EBP*β* promoted NEAT1_1 to release into the cytoplasm (Figure 5G–I; Figure S4C, Supporting Information).

Unlike NEAT1_2, NEAT1_1 is not an essential component of paraspeckles, although it localizes in the nucleus. Therefore, we hypothesized that NEAT1_1 might interact with certain paraspeckle exclusive nuclear proteins, which are downregulated in non‐LSCs, thus lead to partial NEAT1_1 release into the cytoplasm. To test the hypothesis, we performed an RNA pulldown assay, and among the top 10 NEAT1_1‐interacting candidates, we found that NAP1L1 was the only known paraspeckle exclusive nuclear protein (Figure 5J; Table S1, Supporting Information). RNA immunoprecipitation further confirmed that NEAT1_1 but not NEAT1_2 interacted with NAP1L1 (Figure 5K; Figure S4D, Supporting Information). Analysis of GEO database GSE76008 showed that NAP1L1 was downregulated in non‐LSCs compared to LSCs (Figure 5L). Consistently, we observed that NAP1L1 expression level was much lower in CD34^−^ AML cells than CD34^+^ LSCs freshly isolated from AML patients (Figure 5M), so as in the differentiated cells compared with the parent NB4 cells (Figure S4B, Supporting Information). When we knocked down NAP1L1 in NB4 cells with two independent shRNAs (Figure S4E, Supporting Information) and induced the cell for differentiation to examine NEAT1_1 cellular location, we observed that NEAT1_1 was significantly enriched in the cytoplasm (Figure 5N–P; Figure S4F, Supporting Information). In contrast, ectopic NAP1L1 expression suppressed NEAT1_1 releasing from the nucleus and decreased AML cell differentiation (Figure S4G‐L, Supporting Information). Together, it is likely that C/EBP*β* and NAP1L1 co‐operate to elevate cytoplasmic NEAT1_1 in non‐LSCs.

### 2.7 NEAT1_1 Suppresses Wnt Signaling through Interaction with DVL2 and Trim56

**Figure 6:**
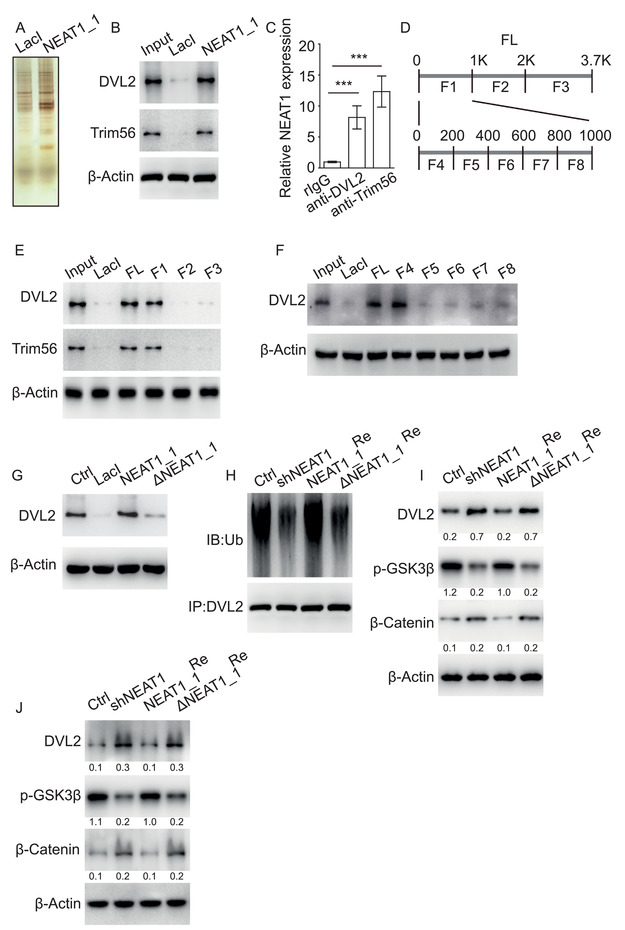
NEAT1_1 suppresses Wnt signaling by interaction with DVL2 and Trim56. A,B) RNA pulldown followed by Western blot showing NEAT1_1 interacting with DVL2 and Trim56 in NB4 cells. C) RNA immunoprecipitation (RIP) showing NEAT1 interacting with DVL2 and Trim56 in NB4 cells. D) Schematic diagram of NEAT1_1 full‐length and truncated fragments. E,F) Mapping analysis of NEAT1_1 fragment interacting with DVL2 and Trim56. G) Western blot following RNA pulldown showing DVL2 interacting with NEAT1_1, but not NEAT1_1 lacking 0–200 bp sequence (ΔNEAT1_1). H) Ubiquitination levels of DVL2 in NB4 cells (Ctrl), NB4 cells with NEAT1 knockdown (shNEAT1), and shNEAT1 cells restored with NEAT1_1 (NEAT1_1^Re^), or ΔNEAT1_1 (ΔNEAT1_1^Re^). I,J) Western blot showing the expression of Wnt signaling components regulated by NEAT1_1 in NB4 cells (I) and human primary LSCs (J). Western blot has been repeated at least three times. Data represent the mean ± SD. *P*‐value was calculated based on a Student's *t*‐test. *p < 0.05; **p < 0.01; ***p < 0.001.

We then explored the mechanism underlying how NEAT1_1 regulates AML and LSCs. We performed RNA pulldown to search for potential NEAT1_1 associated proteins, finding that cytoplasmic disheveled 2 (DVL2) and E3 ubiquitin ligase Trim56 interacted with NEAT1_1 in both human and murine AML cells (**Figure** **6**A,B; Figure S5A, Supporting Information). The interaction of NEAT1_1 with DVL2 and Trim56 were further validated by RNA immunoprecipitation (RIP) (Figure 6C; Figure S5B, Supporting Information). We constructed a series of NEAT1_1 truncated mutants to map the fragments interacting with DVL2 and Trim56 (Figure 6D; Figure S5C, Supporting Information). We found that the fragment F1 (0 to 1 kb) of NEAT1_1 bound to DVL2 and Trim56 (Figure 6E; Figure S5D, Supporting Information). We further truncated the fragment F1 into 5 smaller fragments and found only the fragment F4 (0 to 200 bp) interacted with DVL2 (Figure 6F; Figure S5E, Supporting Information), while all the 5 smaller truncates interacted with Trim56 (Figure S5F, Supporting Information). We then generated a truncated NEAT1_1 in which only fragment F4 was deleted (ΔNEAT1_1). RNA pulldown confirmed that ΔNEAT1_1 failed to bind to DVL2 (Figure 6G; Figure S5G, Supporting Information).

DVL2 is a key regulator of Wnt signaling, which stabilizes *β*‐catenin by disassembling the degradation complex APC/Axin/CK1*α*/GSK3*β*.^[^ ^8^ ^]^ In addition, DVL2 expression level is tightly associated with Wnt activity and tumor progression.^[^ ^26^, ^27^ ^]^ Trim56 is a E3 ubiquitin ligase and has been reported to target various proteins as well as regulate tumor progression.^[^ ^27^ ^]^ Therefore, we hypothesize that cytoplasmic NEAT1_1 associates with DVL2 and Trim56, resulting in degradation of DVL2 and suppression of Wnt activity. To test the hypothesis, we knocked down total NEAT1 and restored with NEAT1_1 or ΔNEAT1_1 in human AML cell NB4 and murine AML cell C1498. We observed that compared to control cells, total NEAT1 knockdown suppressed DVL2 ubiquitylation, which was reverted by restoring NEAT1_1, but not ΔNEAT1_1 (Figure 6H; Figure S5H, Supporting Information). In addition, we found that total NEAT1 knockdown upregulated DVL2 expression and enhanced Wnt activity in AML cells (Figure 6I; Figure S5I, Supporting Information). Conversely, restoration of NEAT1_1, but not ΔNEAT1_1 suppressed DVL2 expression and Wnt activity (Figure 6I; Figure S5I, Supporting Information). To further verify that the ubiquitination degradation of DVL2 is caused by Trim56, we ectopically expressed Trim56 in NB4 cells, NB4 cells with NEAT1 knockdown and NEAT1 knockdown cells restored with NEAT1_1 or ΔNEAT1_1. We found that overexpression of Trim56 increased DVL2 ubiquitination and decreased DVL2 expression in the control cells, but not in the NEAT1 knockdown cells. In addition, restoring NEAT1_1, but not the ΔNEAT1_1 rescued the phenotype of Trim56 overexpression (Figure S5J,K, Supporting Information). We further knocked down total NEAT1 and restored with NEAT1 or ΔNEAT1_1 in CD34^+^ LSCs isolated from AML patients. Consistently, we observed that restoring NEAT1_1 expression reverted DVL2 expression and Wnt activity elevated by total NEAT1 knockdown (Figure 6J). In contrast, restoring ΔNEAT1_1 had no influence on DVL2 expression and Wnt activity (Figure 6J). To investigate whether NEAT1_1 regulates Wnt target gene expression, we measured the expression of several LSC‐related Wnt target genes by RT‐qPCR and *β*‐catenin complex binding to the target genes by ChIP‐qPCR in NB4 cells with NEAT1 knockdown and restored with NEAT1_1 or ΔNEAT1_1. We observed that NEAT1 knockdown upregulated the expression of the target genes and the binding of *β*‐catenin complex on the target gene promoters, which was abrogated by restoring NEAT1_1, but not ΔNEAT1_1 (Figure S5L‐N, Supporting Information). We then ectopically expressed NEAT1_1 alone or NEAT1_1 and *β*‐catenin together in NB4 cells. Colony formation assay showed that overexpression NEAT1_1 alone suppressed colony formation efficiency, while overexpression of *β*‐catenin efficiently rescued the phenotype caused by NEAT1_1 overexpression (Figure S5O, Supporting Information). Together, the data show that NEAT1_1 suppresses Wnt signaling through association with DVL2 and Trim56.

### 2.8 NEAT1_1 Suppresses AML Progression and LSC Function through Manipulating Wnt Signaling

**Figure 7:**
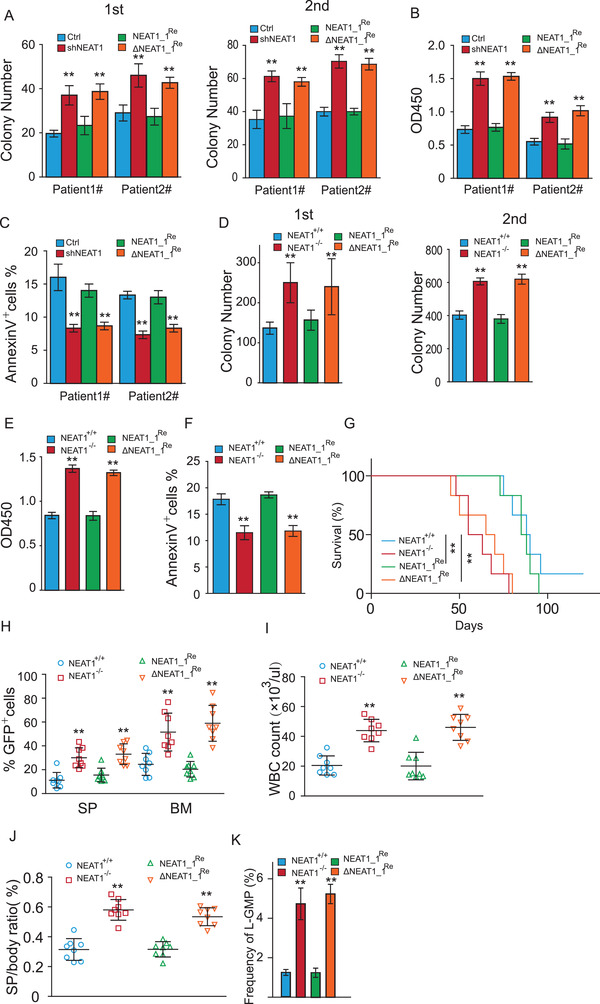
NEAT1_1 suppresses leukemogenesis through inactivating Wnt signaling. A–C) Colony formation (A), proliferation (B), and apoptosis (C) of primary CD34^+^ LSCs from diagnostic (#1) and relapsed (#2) AML patients when total NEAT1 was knocked down (shNEAT1), restored with NEAT1_1 (NEAT1_1^Re^), or the 0–200 bp sequence (ΔNEAT1_1^Re^) was restored. D–F) Colony formation (D), proliferation (E), and apoptosis (F) of primary lin^−^ cells transducing with FLT3‐ITD‐GFP. Lin^−^ cells were isolated from wild‐type (NEAT1^+/+^) and NEAT1 knockout (NEAT1^−/−^) murine bone marrow, and restored with NEAT1_1 (NEAT1_1^Re^) or NEAT1 lacking 0–200 bp sequence (ΔNEAT1_1^Re^). G) Kaplan–Meier survival plots of bone marrow transplantation recipient mice (*n* = 6, each group). H) Percentage of GFP positive cells in spleen (SP) and bone marrow (BM) of recipient mice transplanted with FLT3‐ITD‐GFP‐transformed NEAT1^+/+^, NEAT1^−/−^, NEAT1_1^Re^, and ΔNEAT1_1^Re^ lin^−^ cells (*n* = 8, each group). I) White blood cell (WBC) count in peripheral blood (PB) of recipient mice (*n* = 8, each group). J) Spleen weight of recipient mice (*n* = 8, each group). K) Percentage of L‐GMP cells in recipient mice (*n* = 8, each group). The data represent the mean ± SD in (A–F) and mean ± S.E.M. in (H–K). *P*‐value was calculated based on log‐rank test in (G) and a Student's *t*‐test in other figures. *p < 0.05; **p < 0.01.

To access whether NEAT1_1 regulates LSC and leukemogenesis through NEAT1_1‐mediated DVL2 degradation, we knocked down total NEAT1 and restored NEAT1_1 or ΔNEAT1_1 in NB4 and THP‐1 cells (Figure S6A, Supporting Information). We observed that restorage of NEAT1_1 reverted total NEAT1 knockdown‐elevated colony formation, proliferation, and differentiation (Figure S6B–D, Supporting Information). In contrast, restoring ΔNEAT1_1 failed to rescue the phenotypes resulting from total NEAT1 knockdown (Figure S6B–D, Supporting Information). We then performed similar assays on CD34^+^ LSCs freshly isolated diagnostic or relapse AML patients (Figure S6E, Supporting Information). Consistently, we observed that total NEAT1 knockdown increased colony formation and proliferation, and decreased apoptosis, which was reverted by restoring NEAT1_1, but not ΔNEAT1_1 (**Figure** **7**A–C). We then conducted FLT3‐ITD‐induced murine AML model by restoring NEAT1_1 or ΔNEAT1‐1 into NEAT1^−/−^ Lin^−^ murine bone marrow cells. We observed that knockout of NEAT1 increased Wnt activity through suppression of DVL2 ubiquitination, which was rescued by restoring NEAT1_1, but not ΔNEAT1_1 (Figure S6F,G, Supporting Information). Furthermore, restoring NEAT1_1, but not ΔNEAT1_1 suppressed colony formation, cell proliferation, and apoptosis (Figure 7D–F). We further performed the bone marrow transplantation assay, finding that NEAT1_1, but not ΔNEAT1, could revert NEAT1^−/−^‐accelerated AML progression and mice death (Figure 7G). NEAT1^−/−^‐increased donor cell ratio, white blood cell count, spleen weight, and L‐GMP frequency in the receipt bone marrow (Figure 7H–K). Taken together, the data show that NEAT1_1, but not truncated NEAT1_1 lacking DVL2 interacting sequence, suppresses AML progression and LSC function, suggesting that NEAT1_1‐mediated DVL2 degradation is critical for leukemogenesis.

## 3 Discussion

NEAT1 has long been considered as an oncogene that promotes various solid tumor progression. Here, we show a contrary phenomenon, that NEAT1 functions as a tumor suppressor rather than an oncogene in AML, suppressing leukemogenesis while having no significant effects on normal hematopoiesis. AML is a heterogeneous disease, harboring a number of genetic aberrations, thereby making AML treatment challenging. We found that NEAT1 was downregulated in various subtypes of AML with a complex karyotype and various genetic aberrations, and that decreased NEAT1 expression was correlated with recurrence in AML patients. Additionally, we found that deletion of NEAT1 accelerated AML progression in multiple murine AML models, mediated by MLL‐AF9, HOXA9‐Meis1, or FLT3‐ITD. We further demonstrated that NEAT1 could regulate both diagnostic and recurrent AML progression. These evidences suggest that NEAT1 effects fundamental AML development and might be broadly used for treatment of AML with multiple genetic abnormalities.

LSCs are considered as the fundamental cause of AML initiation, progression and relapse. Our findings revealed that NEAT1 enhanced LSC differentiation through inactivating Wnt signaling, which plays a critical role in tumor initiation and progression. Emerging evidence has shown that lncRNAs were involved in manipulating Wnt signaling, while the regulatory effects mainly occur in the nucleus, through interactions with transcription factors or participation in chromatin remodeling^[^ ^28^ ^]^ or LncRNAs act as sponges to protect Wnt components from micRNAs‐mediated suppression.^[^ ^29^ ^]^ In our study, we demonstrated that NEAT1 interacted with Trim56 and DVL2 in the cytoplasm, inactivating Wnt signaling through enhancing Trim56‐mediated DVL2 degradation. DVL2 is a key Wnt component that activates Wnt signaling. Thus, targeting DVL2 by applying Trim56 or DVL2‐interacted NEAT1 truncation may be a potential strategy to treat AMLs.

NEAT1_2 is an essential component of paraspeckles, regulating various cellular processes in paraspeckle dependent manner. In contrast, NEAT1_1 may localize in non‐paraspeckle foci, suggesting that NEAT1_1 may have a distinct role other than its function in paraspeckles. However, the exact role of NEAT1_1 had remained largely unknown. In this study, we observed that NEAT1 was downregulated in LSCs compared to non‐LSCs. Furthermore, NEAT1 deficiency promoted leukemogenesis through enhancing LSC self‐renewal, while restoring NEAT1_1significantly recused total NEAT1 deletion‐caused enhancement of leukemogenesis and LSC self‐renewal. The observation suggests that NEAT1_1 essentially regulates AML progression. Notably, we found that both NEAT1_1 and NEAT1_2 were downregulated in LSCs. However, it remains unclear how NEAT1_2 contributes to leukemogenesis, which provides a technical challenge to generate a NEAT1_2 specific knockout mouse. In addition, we found that NEAT1_1 was released from the nucleus to cytoplasm during LSC differentiation, a process regulated by transcription factor C/EBP*β* and nuclear protein NAP1L1. Consistent with NEAT1_1 translocation, the expression of C/EBP*β* is upregulated and NAP1L1 is downregulated during LSC differentiation. Collectively, we show that NEAT1_1 is translocated into the cytoplasm and suppresses leukemogenesis and LSC self‐renewal.

## 4 Experimental Section

### Mice and Animal Housing

The NEAT1 KO mouse was a gift from Prof. Mian Wu of the University of Science and Technology of China. B6.SJL (CD45.1) mice were a gift from Prof. Mingzhao Zhu of the Institute of Biophysics, Chinese Academy of Sciences. C57BL/6 and NSG mice were purchased from GemPharmatech Corporation. All laboratory mice were maintained in the animal facility at the Institute of Biophysics, Chinese Academy of Sciences. All mouse maintenance and procedures were approved by the Biomedical Research Ethics Committee of the Institute of Biophysics, Chinese Academy of Sciences, and were performed according to the relevant ethical regulations regarding animal research. All studies were approved by the Ethics Committee of the Institute of Biophysics, Chinese Academy of Sciences (SYXK2019010).

### AML Patient Samples and Normal Hematopoietic Cell Samples

The fresh AML patient samples were obtained from the Fifth Medical Center of Chinese PLA General Hospital with informed consent from all donors. All studies were approved by the Ethics Committee of the Fifth Medical Center of Chinese PLA General Hospital and the Institute of Biophysics, Chinese Academy of Sciences (ky‐2018‐6‐42). AML patient information are shown in Table S2, Supporting Information. Blasts and mononuclear cells were purified using Ficoll‐Paque PLUS (GE healthcare Life Sciences) and the lineage negative cells (lin^–^) were enriched with a human lineage cell depletion kit (130‐092‐211, Miltenyi). Lin^–^CD34^+^ LSCs, or normal lin^–^CD34^+^ hematopoietic stems were sorted by FACS using FITC‐anti‐CD34 (eBiosense).

### Cell Culture

Human AML cell THP1, NB4 and murine AML cell C1498 were obtained from America Type Culture Collection (ATCC) and cultured in RPMI‐1640 (RPMI = Roswell Park Memorial Institute medium) supplemented with 10% fetal bovine serum (FBS) and 1% penicillin–streptomycin. For differentiation, THP‐1 cells were treated with 100 ng mL^−1^ phorbol myristate acetate (PMA) for 24 h and NB4 cells were treated with 1 × 10^−6^ m all‐*t* *rans* retinoic acid (ATRA) for 3 days. HEK 293T cells were cultured in Dulbecco's modified Eagle's medium (DMEM) supplemented with 10% FBS and 1% penicillin–streptomycin. Primary cells were cultured in RPMI‐1640 supplemented with 10% FBS, SCF (100 ng mL^−1^), TPO (100 ng mL^−1^), FLT3L (100 ng mL^−1^), IL‐3 (10 ng mL^−1^), and IL‐6 (10 ng mL^−1^) (Pero Tech).

### Colony‐Forming Assay

Colony‐forming assay was performed as previously described.^[^ ^7^ ^]^ Briefly, human primary cells were plated in methyl‐cellulose medium at the density of 20 000 cells per well in 24‐well plates (MethoCult H4434; Stem Cell Technologies). Mouse BM cells were cultured in methylcellulose medium at the density of 60 000 cells per well in12‐well plates (MethoCultTM M3434, Stem Cell Technologies). Leukemia cell line THP1 and NB4 were plated in 1.2% methylcellulose medium at a density of 100 000 cells per well in 6‐well plates. Colonies were evaluated and scored after 7–10 days of incubation. Serial replating was performed by collecting colony cells and replating them into new dishes with methylcellulose medium after digestion. Colony numbers were counted and compared for each passage.

### Cell Proliferation and Apoptosis Assays

The cell proliferation assay was conducted using the cell counting kit‐8 (HY‐K0301, MCE) following the manufacturer's instruction. Briefly, the cells were seeded into a 96‐well plate in triplicates at the density of 5000–8000 cells/100 µL. After 48 h, 10 µL dye solution was added and incubated at 37 °C for 3–4 h. The absorbance at 450 nm was measured using Mark microplate absorbance reader (Bio‐Rad). APC Annexin V apoptosis Detection kit (AO2001‐11P‐H, Tianjin Sungene Biotech Co., Ltd) was used for apoptosis assays following the manufacturer's instruction.

### Plasmid Construction

NEAT1_1, ΔNEAT1_1, C/EBP*β*, NAP1L1, Trim56 and *β*‐catenin were cloned into the pCDH lentiviral vector. Plasmid pMSCV‐FLT3‐ITD‐Y591F/Y919F, pMSCV‐FLAG‐MLL‐AF9, and pTY‐EF1a‐Hoxa9‐p2a‐Meisl‐p2a‐GFP were purchased from Addgene (#74499, #71444, #61738) and subcloned into the pMSCV PIG vector to achieve FLT3‐ITD‐GFP, MLL‐AF9‐GFP, and HOXA‐Meisl‐GFP construct, respectively. The shRNAs targeting human NEAT1, NAP1L1, or mouse NEAT1 were cloned into pLKO.1. shRNA sequences are shown in Table S3, Supporting Information.

### Lentivirus Preparation, Precipitation, and Infection

Lentiviral constructs were transfected into HEK293T cells together with helper plasmids pMD2.G and psPAX2 (Addgene) using Lipofectamine 2000 (Invitrogen). After 48 and 72 h of transfection, the viral particles were harvested, concentrated, and used to infect the target cells in the presence of 8 µg mL^−1^ Polybrene. Spinoculation was then conducted at 1600 rpm for 60 min. The positively infected cells were selected with 1 µg mL^−1^ puromycin, 1 µg mL^−1^ hygromycin or fluorescence.

### Murine and Human Leukemia Model

For the murine leukemia model, bone marrow cells were extracted from 8‐week‐old wild‐type and NEAT1 knockout mice. Lineage negative cells (lin^−^) were enriched with a hematopoietic stem/progenitor cell enrichment kit (130‐090‐858, Miltenyi), and infected with FLT3‐ITD‐GFP, MLL‐AF9‐GFP, or HOXA‐Meisl‐GFP viral particles in the presence of 8 µg mL^−1^ Polybrene. The transformed cells (2 × 10^5^ to 3 × 10^5^) were transplanted into lethally (8.5 Gy) irradiated B6.SJL (CD45.1) mice by tail‐ vein injection. For the 2nd transplantation, 2000 GFP^+^ leukemia cells isolated from bone marrow of the 1st transplanted mice were transplanted into sub‐lethally (4.5 Gy) irradiated B6.SJL (CD45.1) mice by tail‐vein injection. For the human leukemia model, 2 × 10^5^ infected AML patient‐derived lin^−^ CD34^+^ cells were transplanted into sub‐lethally irradiated (2.0 Gy) 10‐week‐old NSG mice by tail‐vein injection.

### Flow Cytometry Analysis

In normal hematopoiesis assay, after removal of red blood cells with RBC lysis buffer, the BM cells were first stained with biotin‐conjugated anti‐CD4, anti‐CD11b, anti‐CD8a, anti‐CD3e, anti‐Gr‐1, anti‐B220, together with APC‐anti‐c‐Kit, PE‐anti‐Sca‐1, AF700‐anti‐CD34, Percp‐eFluor710‐anti‐CD135 for stem cells, or together with APC‐anti‐c‐Kit, PE‐anti‐Sca‐1, AF700‐anti‐CD34, PE‐Cy7‐anti‐CD16/32, Percp‐Cy5.5‐anti‐CD127 for progenitor cells. eFluor450‐conjugated Streptavidin was then applied to recognize biotin‐conjugated antibodies. For analysis of mature cells, the cells were stained with FITC‐anti‐CD4, PE‐Cy7‐anti‐CD8, APC‐anti‐CD11b, APC‐Cy7‐anti‐Gr‐1, PE‐anti‐B220. To distinguish CD45.2^+^ donor‐derived cells, AF700‐anti‐CD45.2 and BV650‐anti CD45.1 were used. To analyze LSCs in BM, red blood cells were removed by RBC lysis buffer and the remaining cells were first stained with biotin‐conjugated anti‐CD4, anti‐CD11b, anti‐CD8a, anti‐CD3e, anti‐Gr‐1, anti‐B220, together with APC‐anti‐c‐Kit, PE‐anti‐Sca‐1, AF700‐anti‐CD34, Percp‐Cy5.5‐anti‐CD127, PE‐Cy7‐anti‐CD16/32. Streptavidin‐conjugated eFluor450 was then applied to recognize biotin‐conjugated antibodies. All flow cytometric analysis and cell sorting were performed on BD FACSAria III or BD LSRForsseta Flow Cytometer in the Core Facility of the Institute of Biophysics, Chinese Academy of Sciences and the data were analyzed with FlowJo software. The vendor and the catalog number of antibodies are listed in Table S4, Supporting Information.

### Single‐Molecule RNA FISH and Fluorescence Intensity Quantification

Probes labeled with Quasar 570 were purchased from Biosearch Technologies (SMF‐2036‐1 and SMF‐1063‐5). RNA FISH was performed as previously described.^[^ ^30^ ^]^ Briefly, cells were fixed by 4% paraformaldehyde (PFA) at room temperature for 10 min, followed by permeabilization with 70% ethanol for 1 h at 4 °C. After incubation with Hybridization buffer (SMF‐HB1‐10, Biosearch Technologies) for 1 h at 37 °C, the cells were incubated with hybridization buffer containing 125 × 10^−9^ m probes in the dark at 37 °C overnight. After stained with DAPI (5 ng mL^−1^), the cells were attached on a clean glass microscope slide and observed under a fluorescent microscope (FV1200, Olympus). Raw images were processed with softWoRx 7.0 and Image J. Deconvolution images were generated by softWoRx 7.0. Nuclear and cytoplasmic fluorescence intensity was quantified using the “JACoP” plugin for Image J.

### In Vitro RNA Transcription and Purification

Fragments of 200 ng PCR‐amplified T7‐DNA were incubated with 1 µL T7 RNA polymerase enzyme and 1 × 10^−3^ m biotin‐labeled dNTP (11685597910, Roache). In vitro transcription was carried out for 14 h at 37 °C, followed by DNase I treatment for 30 min at 37 °C to remove DNA templates. Transcribed RNA was purified using RNA isolate kit (R1015, ZYMO) according to the manufacturer's instruction.

### RNA Pulldown and RNA Immunoprecipitation

NB4 and C1498 cell line were used to perform RNA pulldown and RNA immunoprecipitation. After preclearing with 30 µL streptavidin‐coupled magnetic beads (Invitrogen) for 2 h, cell lysates were incubated with 3 µg biotinylated RNA for 3 h. Streptavidin‐coupled magnetic beads (Invitrogen) were added to the reaction mix to isolate the RNA‐protein complex. The retrieved proteins were collected for mass spectrometry and Western blot. For RNA immunoprecipitation, cell lysates were precleared with 20 µL protein A/G beads, and incubated with 20 µL protein A/G beads coated with indicated antibodies at 4 °C for 2 h. After extracted from the immunocomplexes, RNAs were analyzed by RT‐qPCR.

### Isolation of Cytoplasmic and Nuclear RNA

Cells were rinsed twice with ice‐cold PBS and centrifuged at 1000 rpm for 3 min. Cell pellets were suspended by gentle pipetting in 200 µL lysis buffer (Thermo Scientific NE‐PER) and incubated on ice for 10 min, followed by centrifugation at 13 000 rpm for 3 min at 4 °C. The supernatants were collected carefully into a new tube and RNA was isolated using Trizol reagent. The nuclear pellets underwent additional washing with 200 µL lysis buffer, followed by RNA isolation with Trizol reagent. To elevate the efficiency of NEAT1_2, cell lysates in Trizol were heating at 55 °C for 10 min with 1000 rpm agitation by thermomixer before RNA extraction.^[^ ^31^ ^]^

### Chromatin Immunoprecipitation

3 × 10^7^ NB4 cells were rinsed twice with ice‐cold PBS. Protein‐DNA was crosslinked by 1% formaldehyde for 10 min at room temperature. Protein‐DNA complexes were sonicated to generate fragments between 200 and 500 bps in length. The pre‐cleared fragments were incubated with 10 µg of anti‐C/EBP*β* or anti‐*β*‐catenin antibody or IgG (as a negative control) overnight, followed by immunoprecipitation by protein G PLUS‐agarose. The DNA‐protein crosslink was reversed by heating at 65 °C overnight, followed by digestion with proteinase K at 45 °C for 2 h. DNA was then recovered with QIAquick PCR purification kit (#28104, Qiagen). qPCR was performed to measure the binding enrichment of C/EBP*β*.

### RT‐qPCR

RNA was purified using TRIzol (Life Technologies) as previously described and evaluated by agarose gel electrophoresis. cDNA was synthesized using HiScript II Q RT SuperMix(R223, Vazyme). qPCR was performed using Fast SybrGreen PCR Master regents (Q311‐02, Vazyme) on the QuantStudio 3 real‐time PCR instrument Primer sequences are listed in the Table S3, Supporting Information.

### Western Blot Analysis

Cells were lysed in RIPA buffer supplemented with protease inhibitors (04693132001, Roche) and phosphatase inhibitor (4906837001, Roche). Proteins were separated by 10% SDS‐PAGE and transferred to a PVDF membrane. Membranes were blocked with 5% non‐fat milk in PBS for 1 h at room temperature and incubated with primary antibody overnight at 4 °C. After washing with PBST three times, membranes were incubated with horseradish peroxidase (HRP)‐conjugated secondary antibodies for 1 h at room temperature. The target proteins were detected by enhanced chemiluminescence with chemiluminescence imaging system (Tanon).

### Public Database Analysis

TCGA datasets were used for analysis of NEAT1 expression in AML and multiple solid tumors. The GEO dataset (GSE114868) was used for analysis of NEAT1 expression in AML cells and healthy controls. The GEO dataset (GSE63270) was used for analysis of NEAT1 expression in healthy HSCs and AML LSCs. The GEO dataset (GSE13159) was used for analysis of NEAT1 expression levels among multiple types of AML with healthy controls. The GEO dataset (GSE83533) was used for analysis of the correlation between NEAT1 expression and AML recurrence. The GEO dataset (GSE76008) was used for analysis of the expression of C/EBP*β* and NAP1L1 expression in LSCs and non‐LSCs.

### Statistical Analysis

Statistical analyses were performed with Graphpad Prism 7.0 using Student's *t*‐tests and log‐rank test. All graphs show mean ± SD or mean ± S.E.M. as indicated in the figure legends. *P*‐values of less than 0.05 were considered statistically significant. Each experiment was conducted with biological replicates and repeated no less than three times. Mice were randomly allocated to experimental groups.

## Conflict of Interest

The authors declare no conflict of interest.

## Authors Contribution

H.W.Y. and Z.W. contributed equally to this work. H.W.Y. and P.C.B. came up with the concept, designed the experiments, and wrote the manuscript. H.W.Y. and W.Z performed the experiments. Y.S. and L.D.H. provided the patient samples.

## Supporting information

Supporting Information

## Data Availability

Research data are not shared.
