# Supplementary material for: Cytoplasmic NEAT1 Suppresses AML Stem Cell Self‐Renewal and Leukemogenesis through Inactivation of Wnt Signaling
Source: Adv Sci (Weinh). 2021 Oct 5;8(22):2100914. doi: 10.1002/advs.202100914 (PMC8596104; doi:10.1002/advs.202100914)
Supplement: Supplementary file 1 — Supporting Information [file ADVS-8-2100914-s001.pdf]

© 2021 Wiley-VCH GmbH

ADVANCED SCIENCE Open Access

Supporting Information

for *Adv. Sci.,* DOI: 10.1002/advs.202100914

Cytoplasmic NEAT1 Suppresses AML Stem Cell Self-Renewal and Leukemogenesis through Inactivation of Wnt Signaling

Huiwen Yan, Zhi Wang, Yao Sun, Liangding Hu, Pengcheng Bu

Figure S1

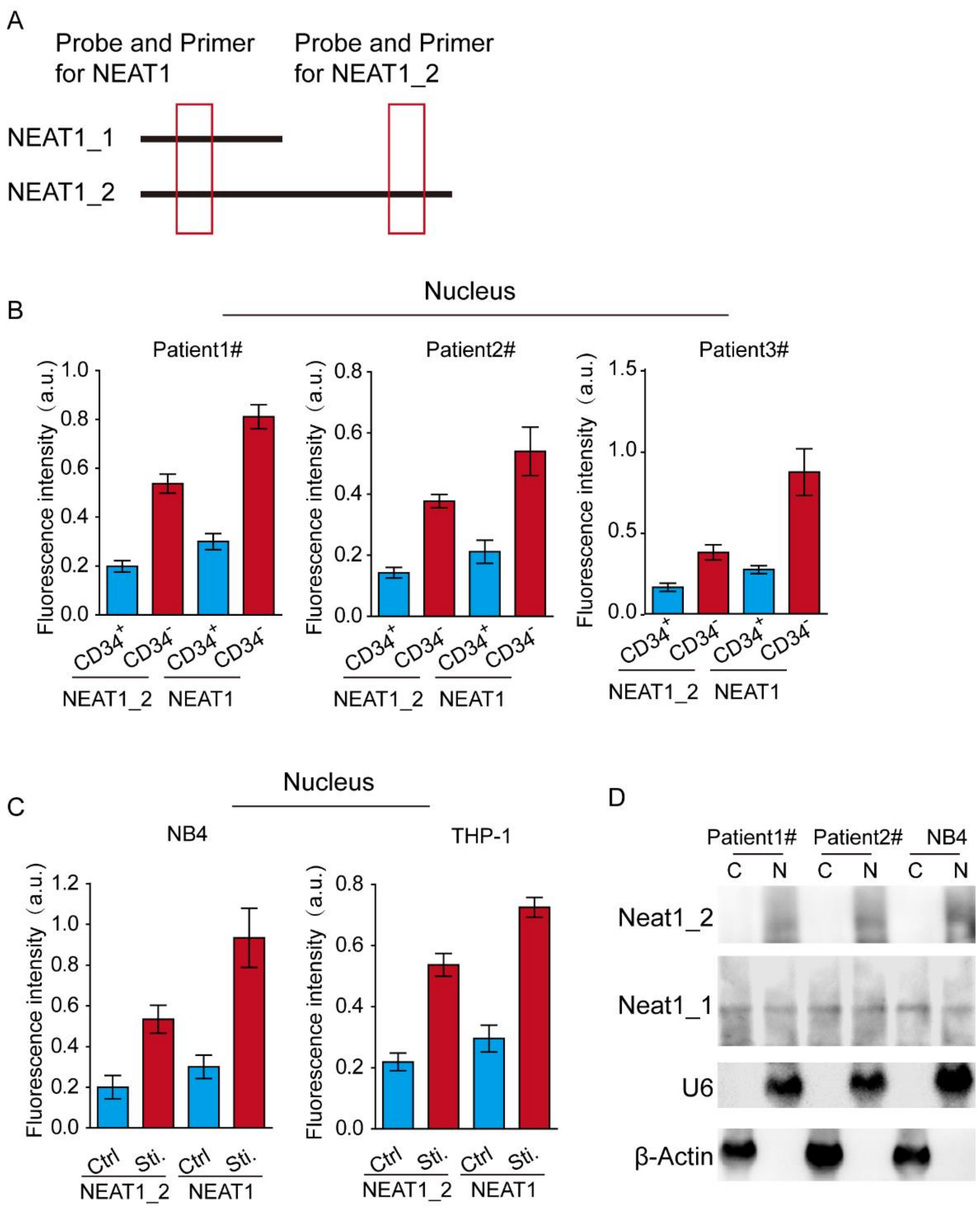

Supplemental Figure 1. NEAT1 is upregulated in differentiated AML cells.

(A) Schematic diagram of probes and primers for NEAT1 and NEAT1_2. **(B)** RNA FISH signals of NEAT1 and NEAT1_2 in the nucleus of primary CD34^{+} and CD34^{-}

cells isolated from bone marrow of AML patients. 20 $CD34^+$ LSCs and 22 $CD34^-$ AML cells were analyzed. **(C)** RNA FISH signals of NEAT1 and NEAT1_2 in the nucleus of NB4 (Ctrl) and ATRA-induced differentiated (Sti) cells, as well THP-1 (Ctrl) and PMA-induced differentiated (Sti) cells. 32 parent and 35 differentiated cells were analyzed. (**D**) Northern blot showing expression of NEAT1_1 and NEAT1_2 in cytoplasm and nucleus isolated from bone marrow cells from AML patients and NB4cells. Actin and U6 are cytoplasmic and nuclear markers, respectively. C: cytoplasm, N: nucleus. Data represent the mean ± SEM. *P*-value was calculated based on student's t-test. *$p<0.05$, **$p<0.01$, and ***$p<0.001$.

Figure S2

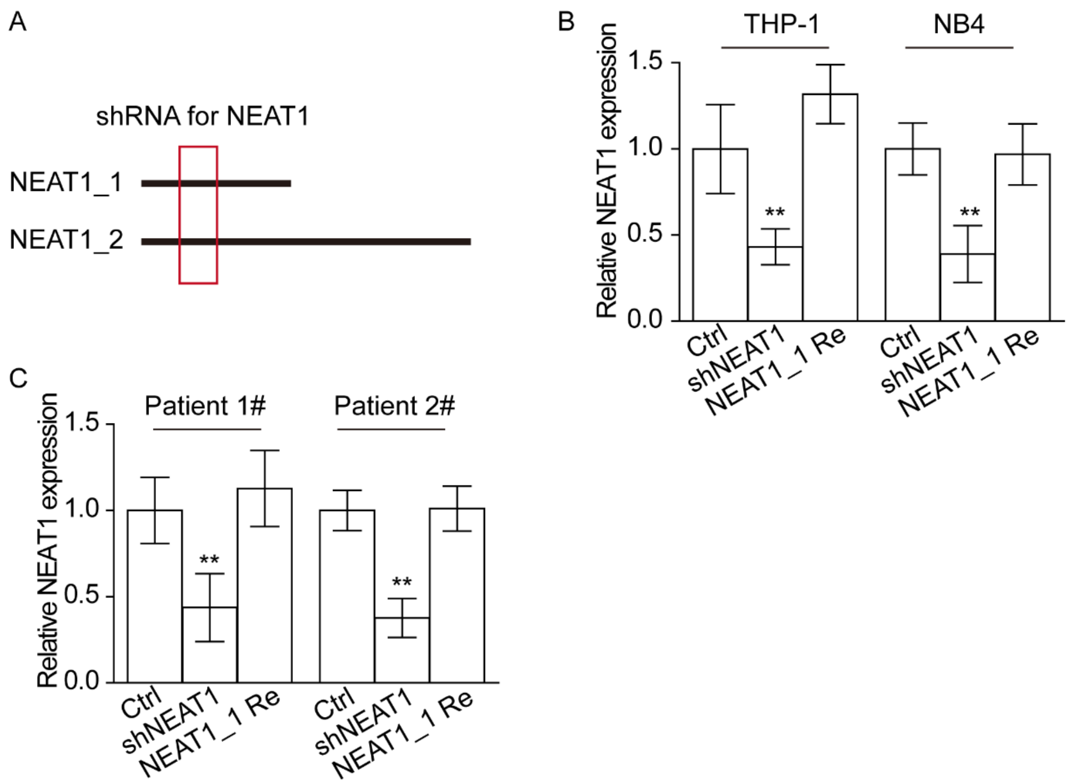

Supplemental Figure 2. Validation of NEAT1 knockdown and rescue.

(A) Schematic diagram of shRNAs for NEAT1 and NEAT1_2. **(B)** RT-qPCR showing NEAT1 knockdown and rescue efficiency in THP-1 and NB4. **(C)** RT-qPCR showing NEAT1 knockdown and rescue efficiency in $CD34^+$ LSCs from AML patients. Data represent the mean ± SD. *P*-value was calculated based on student's t-test. *, $p<0.05$; **, $p<0.01$.

Figure S3

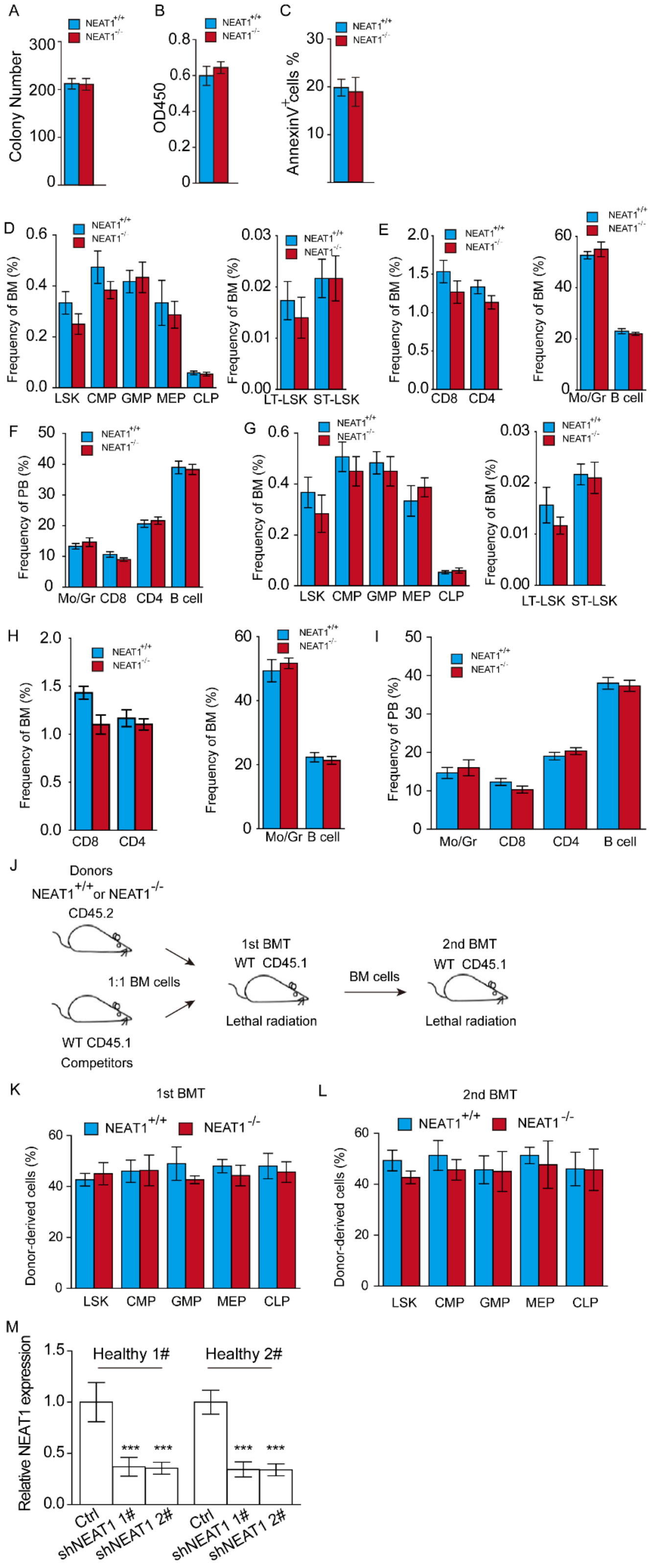

Supplemental Figure 3. NEAT1 is dispensable for normal hematopoiesis.

(A-C) Colony formation (A), proliferation (B), and apoptosis (C) of lin^{-} bone marrow cells isolated wild-type ($NEAT1^{+/+}$) and NEAT1 knockout ($NEAT1^{-/-}$) mice. **(D-F)** Percentage of the population of stem cells, progenitors, and mature lineage cells in the bone marrow and spleen of 1-year-old $NEAT1^{+/+}$ and $NEAT1^{-/-}$ mice (n=6, each group). **(G-I)** Percentage of the population of stem cells, progenitors and mature lineage cell of CD45.2 positive cells in recipient mice 8 months after transplantation (n=6, each group). **(J)** Experimental scheme of the competitive transplantation assays. **(K)** Percentage of donor-derived HSCs and progenitor cell compartments in bone marrow of recipients after primary competitive mixed bone marrow transplantation (n=5, each group). **(L)** Percentage of donor-derived HSCs and progenitor cell compartments in bone marrow of recipients after secondary competitive mixed bone marrow transplantation (n=5, each group). **(M)** NEAT1 knockdown efficiency in human $lin^{-}CD34^{+}$ cells from healthy bone marrow. Data represent the mean ± SD in (M) and mean ± SEM in other Figures. *P*-value was calculated based on student's t-test. *, $p<0.05$; **, $p<0.01$; ***$p<0.001$.

Figure S4

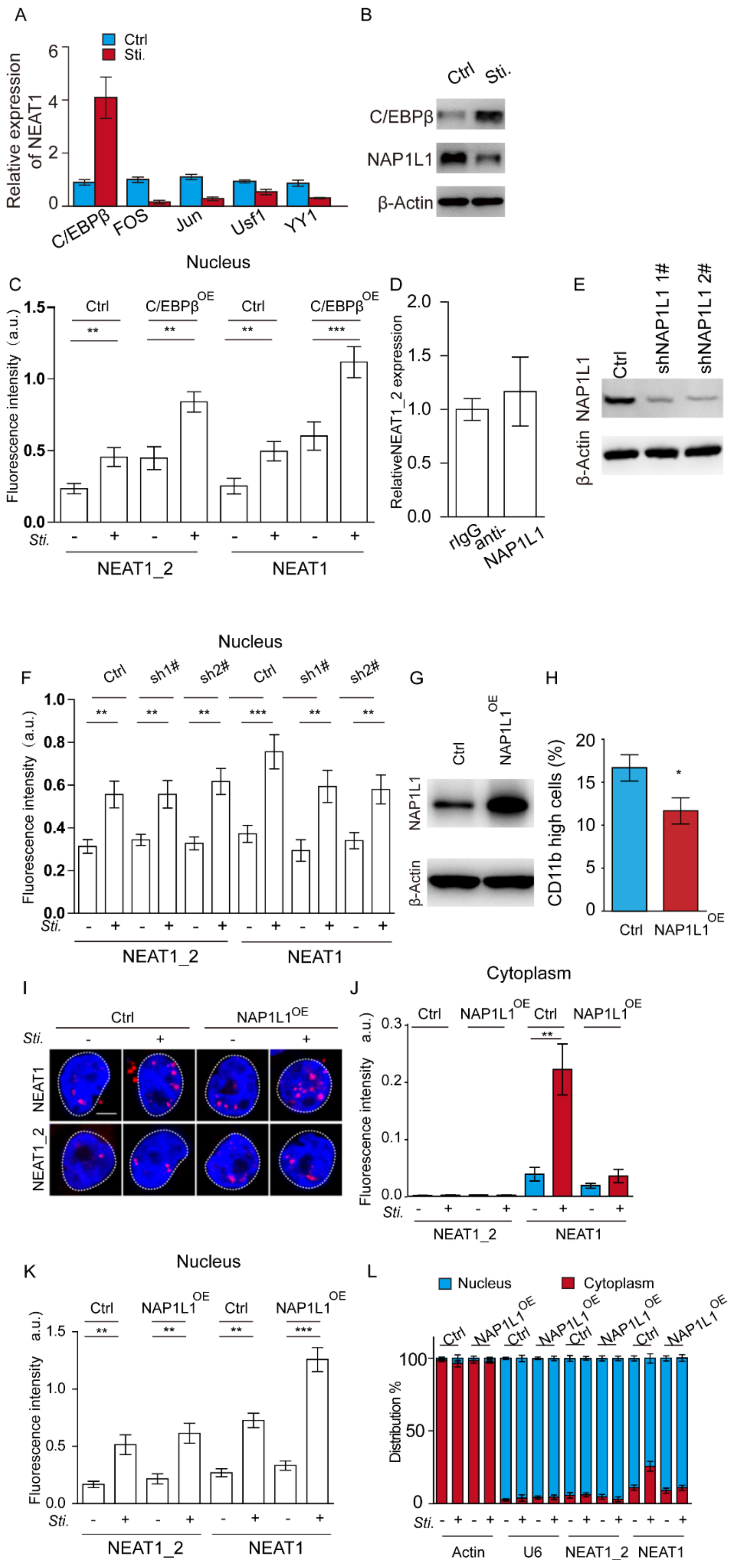

Supplemental Figure 4. C/EBPβ and NAP1L1 upregulates NEAT1_1 in AML cell

cytoplasm.

(A) RT-qPCR showing the expression of the potential transcription factors associated with the 2 kb NEAT1 promoter. **(B)** Western blot showing the expression of C/EBPβ and NAP1L1 in NB4 cells and ATRA-induced differentiated cells. **(C)** RNA FISH signals of nuclear NEAT1 and NEAT1_2 in NB4 cells (Ctrl) and NB4 cells with C/EBPβ-enforced expression (C/EBPβ^{OE}). 35 control cells and 32 C/EBPβ^{OE} cells were analyzed. (**D**) RNA immunoprecipitation (RIP) showing no interaction of NEAT1_2 and NAP1L1 in NB4 cells. **(E)** Western blot showing the knockdown efficiency of NAP1L1. **(F)** RNA FISH signals of nuclear NEAT1 and NEAT1_2 in control (Ctrl) and NAP1L1-knock-down (shNAP1L1 1#, shNAP1L1 2#) NB4 cells. 30 control cells, 32 shNAP1L1 1# cells and 35 shNAP1L1 2# cells were analyzed. (**G**) Western blot showing the NAP1L1 levels in NAP1L1-overexpressed NB4 cells (NAP1L1^{OE}). (**H**) NB4 differentiation in control (Ctrl) and NAP1L1-overexpressed cells (NAP1L1^{OE}). (**I**, **J**) Cellular location of NEAT1 and NEAT1_2 in NB4 cells (Ctrl) and NAP1L1- overexpressed cells (NAP1L1^{OE}). Representative RNA FISH images (I), cytoplasmic RNA FISH signals in each cell (J) and nuclear RNA FISH signals in each cell (K) were shown. 33 control cells and 31 NAP1L1^{OE} cells were analyzed. (**L**) RT-qPCR showing total NEAT1 and NEAT1_2 levels in cytoplasmic and nuclear fractions in NB4 cells (Ctrl) and NAP1L1-overexpressed cells (NAP1L1^{OE}). Actin and U6 are cytoplasmic and nuclear markers, respectively. Data represent the mean ± SD in (A and D) and mean ± SEM in (C, F and J-L). *P*-value was calculated based on student's t-test. *, p<0.05; **, p<0.01; ***, p<0.001.

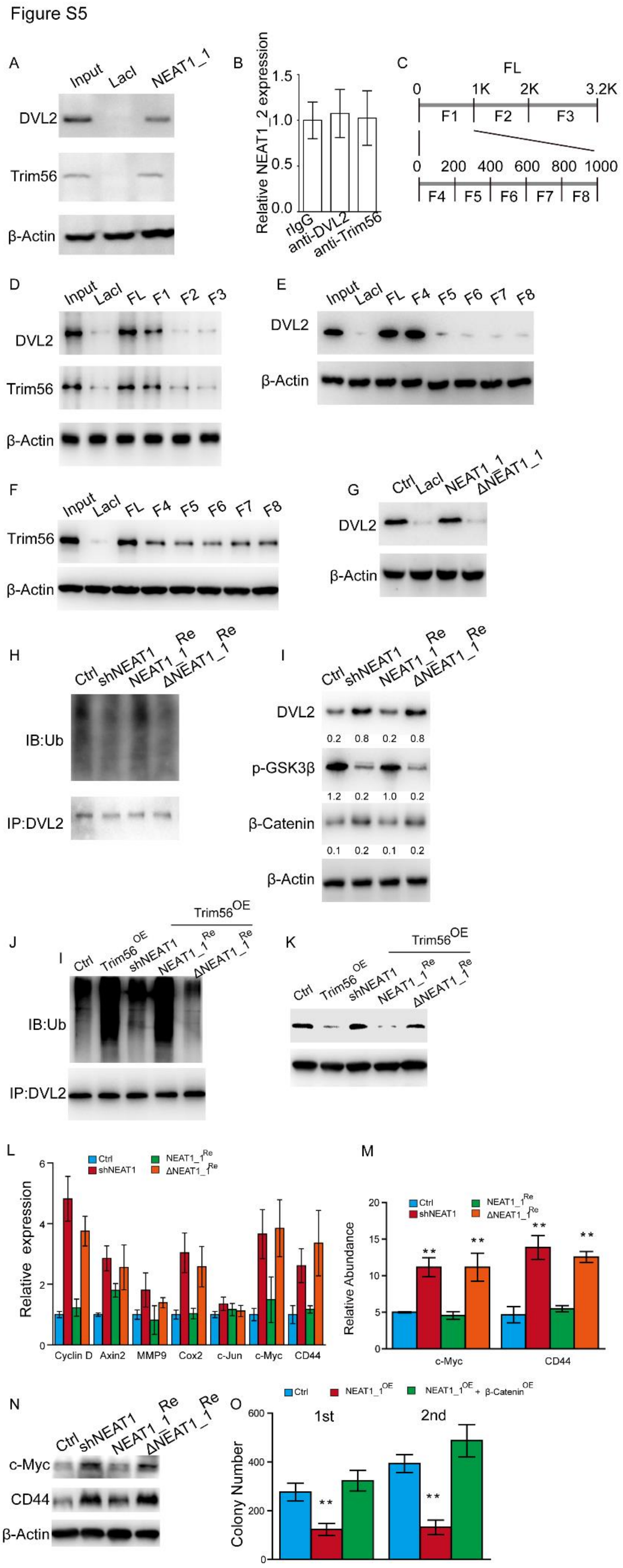

Supplemental Figure 5. NEAT1_1 suppresses Wnt signaling by interaction with

DVL2 and Trim56 in murine AML cells.

(A) RNA pulldown followed by Western blot showing NEAT1_1 interacting with DVL2 and Trim56 in murine AML cell C1498. **(B)** RNA immunoprecipitation (RIP) showing the NEAT1_2 interacting with DVL2 and Trim56 in C1498 cells. **(C)** Schematic diagram of murine NEAT1_1 full-length and truncated fragments. **(D, E)** Mapping analysis of murine NEAT1_1 fragment interacting with DVL2 and Trim56. **(F)** Mapping analysis of NEAT1_1 fragment interacting with Trim56. **(G)** Western blot following RNA pulldown showing DVL2 interacting with NEAT1_1, but not NEAT1_1 lacking 0–200 bp sequence (ΔNEAT1_1). **(H)** Ubiquitination levels of DVL2 in C1498 cells (Ctrl) and C1498 cells with NEAT1 knockdown (shNEAT1), shNEAT1 cells restored with NEAT1_1 ($NEAT1\_1^{Re}$), or ΔNEAT1_1 ($\Delta NEAT1\_1^{Re}$). **(I)** Western blot showing the expression of Wnt signaling components regulated by NEAT1_1 in C1498 cells. Western blot has been repeated at least three times. (**J**) Ubiquitination levels of DVL2 in control (ctrl), Trim56-overexpressed ($Trim56^{OE}$) NB4 cells, Trim56-overexpressed NB4 cells with NEAT1 knockdown, and NEAT1 knockdown cells restored with NEAT1_1 ($NEAT1\_1^{Re}$) or ΔNEAT1_1 ($\Delta NEAT1\_1^{Re}$). (**K**) Western blot showing expression of DVL2 in control (ctrl), Trim56-overexpressed ($Trim56^{OE}$) NB4 cells, Trim56-overexpressed NB4 cells with NEAT1 knockdown (shNEAT1), and NEAT1 knockdown cells restored with NEAT1_1 ($NEAT1\_1^{Re}$) or ΔNEAT1_1 ($\Delta NEAT1\_1^{Re}$). (**L-N**) RT-qPCR (L), ChIP-qPCR (M) and Western blot (N) showing β-catenin target genes in NB4 cells (Ctrl) and NB4 cells with NEAT1 knockdown (shNEAT1), shNEAT1 cells restored

with NEAT1_1 ($NEAT1\_1^{Re}$), or ΔNEAT1_1 ($\Delta NEAT1\_1^{Re}$). (**O**) Colony formation in control (ctrl), NEAT1_1-overexpressed NB4 cells ($NEAT1\_1^{OE}$) and both β-catenin and NEAT1_1-overexpressed NB4 cells ($NEAT1\_1^{OE}$ + β-catenin ^{OE}). Data represent the mean ± SD. *P*-value was calculated based on student's t-test. *, $p<0.05$; **, $p<0.01$; ***, $p<0.001$.

Figure S6

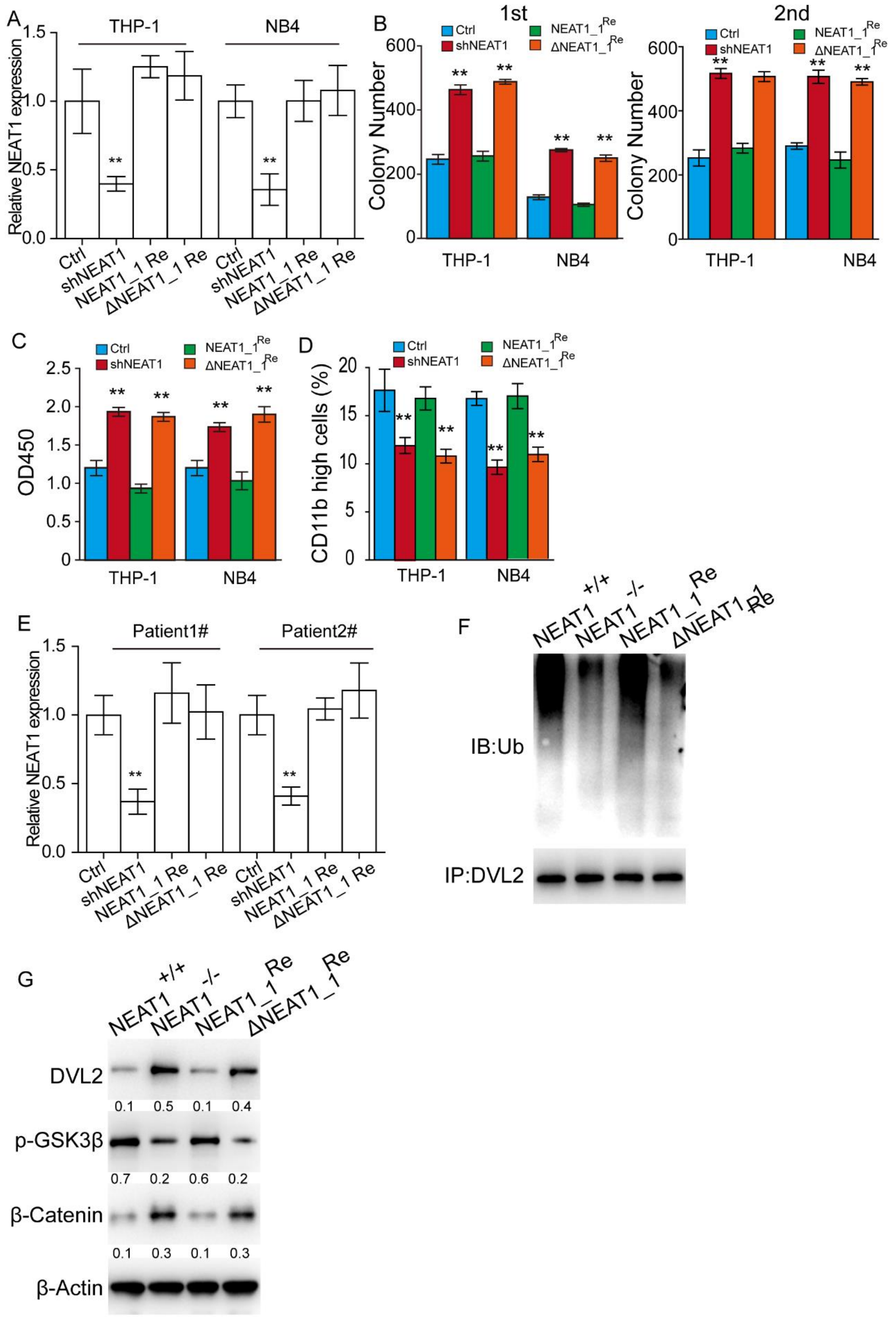

Supplemental Figure 6. NEAT1_1 but not ΔNEAT1_1 regulates AML cell

activities.

(A) RT-qPCR showing the efficiency of NEAT1 knockdown, NEAT1_1 rescue, and ΔNEAT1_1 rescue in THP-1 and NB4 cells. **(B-D)** Colony formation (B), proliferation (C), and differentiation (D) of NB4 and THP1 cells when total NEAT1 was knocked down (shNEAT1) and NEAT1_1 ($NEAT1\_1^{Re}$) or NEAT1 lacking 0–200 bp sequence ($\Delta NEAT1\_1^{Re}$) was restored. **(E)** RT-qPCR showing the efficiency of NEAT1 knockdown, NEAT1_1 rescue, and ΔNEAT1_1 rescue in primary $CD34^+$ LSCs from AML patients. **(F)** Ubiquitination levels of DVL2 in primary $lin^−$ cells transducing with FLT3-ITD-GFP. $Lin^−$ cells were isolated from wild-type ($NEAT1^{+/+}$) and NEAT1 knockout ($NEAT1^{−/−}$) murine bone marrow, and restored with NEAT1_1 ($NEAT1\_1^{Re}$) or NEAT1 lacking 0-200 bp sequence ($\Delta NEAT1\_1^{Re}$). (**G**) Western blot showing the expression of Wnt signaling components in primary $lin^−$ cells transducing with FLT3-ITD-GFP. $Lin^−$ cells were isolated from wild-type ($NEAT1^{+/+}$) and NEAT1 knockout ($NEAT1^{−/−}$) murine bone marrow, and restored with NEAT1_1 ($NEAT1\_1^{Re}$) or NEAT1 lacking 0-200 bp sequence ($\Delta NEAT1\_1^{Re}$). Western blot has been repeated at least three times. Data represent the mean ± SD. *P*-value was calculated based on student's t-test. *, $p<0.05$; **, $p<0.01$; ***, $p<0.001$.

Table S1. List of nuclear proteins pulled down by NEAT1_1

Name	Annotation	Coverage (%)	Ratio of pull down/ctrl	P Value
CNBP	Cellular nucleic acid-binding protein	30	100	<0.0001
SAFB2	Scaffold attachment factor B2	27	100	<0.0001
NONO	Non-POU domain-containing octamer-binding protein	24	100	<0.0001
SFPQ	Splicing factor, proline- and glutamine-rich	15	100	<0.0001
PSPC1	Paraspecklesproteincomponent1	14	100	<0.0001
CPSF	Cleavage and polyadenylation specificity factor subunit	10	100	<0.0001
NAP1L1	Nucleosome assembly protein 1-like 1	8	100	<0.0001
NUBP2	Cytosolic Fe-S cluster assembly factor	8	100	<0.0001
LUC7L	Putative RNA-binding protein Luc7-like 1	3	95	<0.0001
DNAJA2	DnaJ homolog subfamily A member 2	3	95	<0.0001

Table S2. List of patient information

ID	Age	M/F	Source	Diagnostic/Relapsed	Identification
AML1	40	M	Bone marrow	Diagnostic	MLL-AF9
AML2	14	M	Bone marrow	Diagnostic	FLT3-ITD
AML3	42	M	Bone marrow	Diagnostic	Complex
AML4	40	F	Bone marrow	Diagnostic	ASXL1
AML5	52	F	Peripheral blood	Diagnostic	Complex
AML6	28	F	Peripheral blood	Diagnostic	NPM1
AML7	74	F	Bone marrow	Diagnostic	FLT3-ITD
AML8	61	M	Bone marrow	Diagnostic	CBFβ-MYH11
AML9	52	M	Bone marrow	Diagnostic	NRAS, WT1
AML10	29	F	Bone marrow	Diagnostic	Complex
AML11	39	M	Bone marrow	Diagnostic	FLT3-TKD, TP53
AML12	17	M	Bone marrow	Diagnostic	U2AF1, ASXL1
AML13	57	M	Bone marrow	Diagnostic	FLT3-ITD
AML14	67	F	Bone marrow	Diagnostic	ETO
AML15	39	M	Bone marrow	Diagnostic	CSF3R, CEBPA
AML16	51	M	Bone marrow	Diagnostic	FLT3-ITD
AML17	53	M	Bone marrow	Diagnostic	TET2, FLT3-ITD
AML18	26	M	Bone marrow	Diagnostic	NPM1
AML19	50	F	Bone marrow	Diagnostic	FLT3-ITD, NPM1
AML20	50	F	Bone marrow	Diagnostic	NPM1, FLT3-ITD
AML21	61	M	Bone marrow	Diagnostic	CSF3R, ASXL1,
AML22	44	F	Bone marrow	Diagnostic	NRAS, U2AF1
AML23	38	M	Bone marrow	Diagnostic	ETO
AML24	74	F	Bone marrow	Diagnostic	MLL-AF10
AML25	29	M	Bone marrow	Diagnostic	KRAS, TET2
AML26	38	M	Bone marrow	Diagnostic	FLT3-ITD, NPM1
AML27	53	F	Bone marrow	Diagnostic	Complex
AML28	33	F	Bone marrow	Diagnostic	MLL-AF9
AML29	63	M	Bone marrow	Diagnostic	MLL-AF9
AML30	56	M	Bone marrow	Diagnostic	CBFβ-MYH11
AML31	45	M	Bone marrow	Relapsed	NPM1
AML32	31	M	Bone marrow	Relapsed	NRAS
AML33	32	M	Bone marrow	Relapsed	MLL-AF9
AML34	66	F	Bone marrow	Relapsed	FLT3-ITD, NPM1
AML35	46	F	Bone marrow	Relapsed	RUNX1
AML36	65	M	Bone marrow	Relapsed	RUNX1
AML37	57	F	Bone marrow	Relapsed	ETO
AML38	28	F	Bone marrow	Relapsed	NRAS
AML39	61	F	Bone marrow	Relapsed	CEBPA
AML40	72	F	Bone marrow	Relapsed	Complex

Table S3. List of primers and shRNAs

Name	Sequence
H-NEAT1-F	cccttcttcctccctttaact
H-NEAT1-R	cctctcttcctccaccattac
H-NEAT1_2-F	agtagcctctggtgtcatttg
H-NEAT1_2-R	gacacttctccagggaacatac
H-NEAT1promoter-F	gggcgctcttcaaccataaa
H-NEAT1promoter-R	agcttgggtggaatgcttaat
CEBP-F	cgcgacaaggccaagat
CEBP-R	gctgctccaccttcttctg
NAP1L1-F	ccgctcgagatggcagacattgacaac
NAP1L1-R	ccgaccggtgagtgcaagcagcagtga
Trim56-F	ggaagatctatggtttcccacgggtcc
Trim56-R	ccggaattcttaactgtccggagaacg
β-catenin-F	ggaagatctatgcaacgcctggtggcc
β-catenin-R	ccggaattcctagcagtggccggagga
Fos-F	ctgaaggcagaacccctttga
Fos-R	cagtctgctgcatagaaggaa
Jun-F	aatgggcacatcaccactac
Jun-R	tgttctggctatgcagttcag
Usf1-F	cctgtccaaagcctgtgatta
Usf1-R	tccagctgcaactgatctaac
YY1-F	cattgacctctcagaccctaag
YY1-R	acatctttgtgcagcctttatg
Acin-F	ctccatcctggcctcgctgt
Acin-R	gctgtcaccttcaccgttcc
U6-F	gtgctcgcttcggcagcacat
U6-R	ggaacgcttcacgaatttgcg
Sh H-NEAT1-1# S	ccggagccttgtaaatgcctatattctcgagaatataggcatttacaaggcttttttg
Sh H-NEAT1-1# A	aattcaaaaaagccttgtaaatgcctatattctcgagaatataggcatttacaaggct
Sh H-NEAT1-2# S	ccggaaagtttgagttctaaactcactcgagtgagtttagaactcaaacttttttttg
Sh H-NEAT1-2# A	aattcaaaaaaaaagtttgagttctaaactcactcgagtgagtttagaactcaaacttt
Sh M-NEAT1-1# S	ccggaagacaacagggtttgcttatctcgagataagcaaaccctgttgtctttttttg
Sh M-NEAT1-1# A	aattcaaaaaaagacaacagggtttgcttatctcgagataagcaaaccctgttgtctt
Sh M-NEAT1-2# S	aacagtttgggaactaaatagctcgagctatttagttcccaaactgttttttg
Sh M-NEAT1-2# A	aattcaaaaaaaacagtttgggaactaaatagctcgagctatttagttcccaaactgtt
Sh Nap 1#- S	ccggcctattctgaagcacttgaaactcgagtttcaagtgcttcagaataggttttttg
Sh Nap 1#- A	aattcaaaaaacctattctgaagcacttgaaactcgagtttcaagtgcttcagaatagg
Sh Nap 2#- S	ccgggccaagattgaagatgagaaactcgagtttctcatcttcaatcttggcttttttg
Sh Nap 2#- A	aattcaaaaaagccaagattgaagatgagaaactcgagtttctcatcttcaatcttggc

Table S4. List of antibodies

Antibody	Source	Identifier
Anti-human APC-CD45	Biolegend	Cat#368512; RRID: AB_2566372
Anti-human PE-CD11b	Biolegend	Cat#101208; RRID: AB_312791
Anti-human FITC-CD34	Biolegend	Cat#343504; RRID: AB_1731852
Anti-mouse AF700-CD45.2	Biolegend	Cat#109821; RRID: AB_493730
Anti-mouse BV650 CD45.1	Biolegend	Cat# 110735; RRID: AB_11124743
Anti-mouse FITC- CD4	Biolegend	Cat#100405; RRID: AB_312690
Anti-mouse PE-Cy7- CD8	eBioscience	Cat# 25-0081-82; RRID: AB_469584
Anti-mouse APC- CD11b	eBioscience	Cat# 17-0112-82; RRID: AB_469343
Anti-mouse APC-Cy7 Gr-1	eBioscience	Cat# A15424; RRID: AB_2534437
Anti-mouse PE- B220	eBioscience	Cat# 25-0452-82; RRID: AB_469627
Anti-mouse CD4-biotin	Biolegend	Cat# 100404; RRID: AB_312689
Anti-mouse CD11b-biotin	eBioscience	Cat# 13-0112-82; RRID: AB_466359
Anti-mouse CD8a-biotin	Biolegend	Cat# 100704; RRID: AB_31247
Anti-mouse CD3e-biotin	Biolegend	Cat# 100304; RRID: AB_312669
Anti-mouse Gr-1-biotin	Biolegend	Cat# 108404; RRID: AB_313369
Anti-mouse B220-biotin	Biolegend	Cat# 103204; RRID: AB_312989
eFluor450-Streptavidin	eBioscience	Cat# 48-4317-82
Anti-mouse APC-c-Kit	eBioscience	Cat# 17-1172-82; RRID: AB_469433
Anti-mouse PE-Sca-1	eBioscience	Cat# 12-5981-82; RRID: AB_466086
Anti-mouse-AF700-CD34	eBioscience	Cat# 56-0341-82; RRID: AB_493998
Anti-mouse-Percp-eFluor710-Flt3	eBioscience	Cat# 46-4321-82; RRID: AB_10733393
Anti-mouse-PE-Cy7- CD16/32	eBioscience	Cat# 25-0161-82; RRID: AB_469598
Anti-mouse-Percp-Cy5.5- CD127	eBioscience	Cat# 45-1271-42; RRID: AB_1106998
Anti-C/EBPβ	Abcam	Cat# ab32358; RRID: E299
Anti-NAP1L1	Abcam	Cat# ab178687; RRID: 11845
Anti-DVL2	CST	Cat# 3224; RRID: 30D2
Anti-Trim56	Abcam	Cat# 154862; RRID: EPR10583
Anti-Ubquitin	CST	Cat# 3936; RRID: P4D1
Anti-P-GSK3β	CST	Cat# 5558; RRID: D85E12
Anti-β-Catenin	Abcam	Cat# ab32572; RRID: E247
